# Bioinformatic Analysis of C1GALT1 in Cancer: Insights Into Prognosis, Metastasis and Therapeutic Potential

**DOI:** 10.1002/cnr2.70259

**Published:** 2025-06-23

**Authors:** Ecem Kalemoglu, Ayse Caner

**Affiliations:** ^1^ Department of Internal Medicine Rutgers‐Jersey City Medical Center Jersey City New Jersey USA; ^2^ Department of Basic Oncology Institute of Health Sciences, Ege University Bornova Izmir Turkiye; ^3^ Translational Pulmonary Research Center (EGESAM), Ege University Bornova Izmir Turkiye; ^4^ Department of Parasitology Faculty of Medicine, Ege University Bornova Izmir Turkiye

**Keywords:** C1GalT1, cancer, glycosylation, transcriptomics, T‐synthase, tumor‐infiltrating lymphocytes

## Abstract

**Background:**

This study evaluates the expression, regulation, and clinical relevance of C1GALT1, a key enzyme in mucin‐type O‐glycosylation, across a broad spectrum of human cancers. Aberrant glycosylation is a well‐established hallmark of malignancy, contributing to tumor growth, immune evasion, and metastasis. C1GALT1, also known as core 1 β1,3‐galactosyltransferase or T‐synthase, catalyzes the formation of the core 1 O‐glycan structure and requires the chaperone Cosmc for proper folding and activity. While previous studies have implicated C1GALT1 in cancer progression, a systematic pan‐cancer analysis exploring its gene expression patterns, epigenetic regulation, immune interactions, and prognostic significance has not been fully elucidated.

**Aims:**

This study aims to computationally investigate C1GALT1 expression, regulation, and clinical relevance across multiple cancers using TCGA datasets to evaluate its potential as a biomarker and therapeutic target.

**Methods:**

We conducted a comprehensive bioinformatic analysis of C1GALT1 using publicly available datasets from The Cancer Genome Atlas (TCGA). Gene expression, DNA methylation, and survival analyses were performed, along with correlation analyses between C1GALT1 and proliferation‐ or metastasis‐related genes, Cosmc expression, and immune cell infiltration (specifically, regulatory T‐cells [Tregs] and myeloid‐derived suppressor cells [MDSCs]), using transcriptomic web platforms.

**Results:**

C1GALT1 expression was significantly upregulated in gastrointestinal and genitourinary cancers compared to normal tissues, while downregulated in thyroid, breast, and prostate cancers. Elevated expression correlated with reduced overall survival in lung, bladder, liver, and glioma/glioblastoma. DNA methylation analysis showed an inverse correlation between methylation and expression levels in multiple cancer types. C1GALT1 expression positively correlated with Cosmc, proliferation markers (MKI67, PCNA, MCM family, PLK1), and several metastasis‐associated genes. Immune profiling revealed context‐dependent correlations: C1GALT1 negatively correlated with Tregs and MDSCs in gastrointestinal cancers but positively in lung, breast, and prostate cancers.

**Conclusion:**

Our pan‐cancer analysis suggests that C1GALT1 is differentially expressed and epigenetically regulated across tumor types and may contribute to tumor proliferation, metastasis, and immune modulation. While these findings support C1GALT1 as a potential biomarker and therapeutic target, further in vitro and in vivo studies are necessary to validate its mechanistic roles and clinical utility.

## 1 Introduction

Cancer pathogenesis involves intricate cellular mechanisms, notably glycosylation, which refers to modifying proteins and lipids by adding carbohydrate moieties. Glycosylation is a critical biological process that orchestrates the functionality, stability, and interactions of cell surface proteins [1]. This modification occurs via complex pathways involving key components such as glycosyltransferases and glycosidases [2]. Glycosylation can be classified into two principal types based on the bond between the sugar moiety and the amino acid residue: N‐glycosylation and O‐glycosylation [3].

Abnormal glycosylation is a hallmark of many cancers and exerts significant influences on tumor development and progression. Several physiological processes regulated by glycosylation, including cell adhesion, cell‐matrix interactions, epithelial‐to‐mesenchymal transition, tumor growth, invasion, and metastasis, play key roles in cancer progression PEVuZE5vdGU [2, 4]. Therefore, glycans hold promise as clinical biomarkers and therapeutic targets. Aberrant glycosylation results in the formation of tumor‐associated carbohydrate antigens (TACAs), such as truncated O‐glycans (Tn, TF, and sialyl‐Tn antigens), gangliosides, Lewis antigens, and polysialic acid, which are critical in targeted cancer therapies. Numerous clinical tumor biomarkers, including AFP, CA125, CEA, PSA, and CA19‐9, are glycoproteins or glycan‐related entities [2].

C1GalT1, core 1 synthase or T‐synthase, is a glycosyltransferase with its encoding gene located on chromosome 7p14‐7p13. It consists of three exons and catalyzes the transfer of galactose (Gal) from UDP‐Gal to GalNAc and plays a critical role in the O‐glycosylation of proteins, transferring a galactose residue from UDP‐Gal to form T antigen, known as Galβ1‐3GalNAc⍺1‐O‐Ser/Thr [3, 5]. Furthermore, the expression and function of T‐synthase depend on the presence of an endoplasmic reticulum (ER) molecular chaperone known as Cosmc [6]. A deficiency in either T‐synthase or Cosmc leads to impaired elongation of O‐glycans, leading to the expression of the Tn antigen or Sialyl Tn (STn) antigen, an abnormal O‐glycosylation form of T antigen [1, 5, 7]. These Tn and STn antigens are examples of TACAs, with estimates indicating that the Tn antigen is expressed in over 70% of human carcinomas [1].

Loss of T‐synthase or Cosmc due to genetic and epigenetic inactivation may be responsible for Tn expression in human cancer cell lines [8]. Emerging evidence suggests that C1GalT1 fulfills a dual role in cancer, acting in both tumor promotion and suppression. Its tumor‐suppressor effects occur through several pathways, including negative regulation by miR‐181d‐5p and miR‐152, while its deletion can lead to spontaneous gastric and duodenal cancers by disrupting the mucus barrier. On the other hand, the pro‐cancer effects of C1GalT1 are linked to modifications of O‐glycans on downstream targets [3]. Additionally, a recent study has revealed that C1GalT1 is overexpressed at both protein or gene levels in certain prevalent cancers, with this overexpression often correlating with poor prognosis and reduced patient survival [9], leaving its role across different cancers still unclear and in need of further study.

In this study, we aimed to evaluate the role of T‐synthase in cancer pathogenesis by comparing C1GalT1 gene expression levels in healthy and malignant tissues across a spectrum of cancer types by using bioinformatic tools and the TCGA cancer database. Cancer types with statistically significant differences in C1GalT1 gene expression between tumor and normal tissues were further analyzed to determine whether changes in DNA methylation could explain these differences. Additionally, we evaluated patient survival using Kaplan–Meier analysis and compared C1GalT1 gene expression levels between metastatic (M1) and non‐metastatic (M0) cases. Later, to understand the effect of the C1GalT1 gene expression level on tumor proliferation, the expression levels of the C1GalT1 and MKI67 genes were compared across various cancer types, and the relationship between the C1GalT1 expression levels was analyzed. Additionally, the relations between the abundance of tumor‐infiltrating lymphocytes (TILs), especially immunosuppressive regulatory T‐cells (Tregs) and myeloid‐derived suppressive cells (MDSCs), and expression of the C1GALT1 gene were analyzed.

## 2 Materials and Methods

### 2.1 The Human Protein Atlas

The Human Protein Atlas (HPA) (https://proteinatlas.org) was used to compare the C1GalT1 gene expression in different cancer types by using the TCGA datasets [10, 11]. Median Fragments Per Kilobase of transcript per Million mapped reads (FPKM) values were employed for the assessment of gene expression levels.

### 2.2 The UCSC Xena Browser Analysis

The UCSC Xena browser (https://xenabrowser.net/heatmap/) serves as an online platform for visualizing and analyzing functional genomic data pertinent to clinical research. This tool is based on JavaScript [12, 13]. UCSC Xena was used to generate Kaplan–Meier plots comparing overall survival rates between low versus high C1GalT1 gene expression levels. The portal automatically calculates the median gene expression value across all samples, and it divides them into two groups: samples with expression levels above the median as high expression and those with levels below the median as low expression.

The portal was also employed to assess the differences in C1GalT1 gene expression and DNA methylation levels between healthy and malignant tissues across various cancer types, using datasets from the TCGA database. Protocols provided on the UCSC Xena website (https://ucsc‐xena.gitbook.io/project/how‐do‐i) were followed for the analysis.

For the C1GalT1 gene expression, DNA methylation and KM analyses, the TCGA datasets were used as follows (listed alphabetically): bladder cancer—TCGA Bladder Cancer (BLCA); breast cancer—TCGA Breast Cancer (BRCA); cervical cancer—TCGA Cervical Cancer (CESC); colon and rectal cancer—TCGA Colon and Rectal Cancer (COADREAD); endometrioid cancer—TCGA Endometrioid Cancer (UCEC); glioblastoma—TCGA Glioblastoma (GBM); head and neck cancer—TCGA Head and Neck Cancer (HNSC); kidney clear cell carcinoma—TCGA Kidney Clear Cell Carcinoma (KIRC); liver cancer—TCGA Liver Cancer (LIHC); lower grade glioma—TCGA Lower Grade Glioma (LGG); lung cancer—TCGA Lung Cancer (LUNG); melanoma—TCGA Melanoma (SKCM); ovarian cancer—TCGA Ovarian Cancer (OV); pancreatic cancer—TCGA Pancreatic Cancer (PAAD); prostate cancer—TCGA Prostate Cancer (PRAD); stomach cancer—TCGA Stomach Cancer (STAD); testicular cancer—TCGA Testicular Cancer (TGCT); thyroid cancer—TCGA Thyroid Cancer (THCA).

#### 2.2.1 Comparison of the C1GalT1 Gene Expression Levels

C1GalT1 gene expression levels were compared by the UCSC Xena portal. After selecting the corresponding study of TCGA for the cancer type, for the “Genomic” data type, the “C1GALT1” gene and the “Gene Expression” dataset were selected. For the “Phenotype” data type “sample_type” was selected which includes data for tumor and normal tissue samples. If there were any samples from metastatic tissue, recurrent tumor tissue, or any additional tissues, those samples were removed. Later, samples with null values were removed. For the analysis, in the “Chart & Statistics” icon, the “Compare Subgroups” function was employed. For the visualization, a box plot was utilized. For the gene expression comparisons, median gene expression values which were calculated as log2 (normalized counts+1) were used.

#### 2.2.2 Comparison of the C1GalT1 DNA Methylation Levels

The average DNA methylation levels of the C1GalT1 gene were compared using the UCSC Xena portal. Similar to the previous analysis, the appropriate TCGA study for the cancer type was selected, followed by the selection of the “C1GALT1” gene and the “Methylation450k” dataset under the “Genomic” data type. For the “Phenotype” data type, the “sample_type” was selected, which includes data for tumor and normal tissue samples. If there were any samples from metastatic tissue, recurrent tumor tissue, or any additional tissues, those samples were removed. Later, samples with null values were removed. Finally, for the methylation levels, the “Gene average” function was used. For the analysis in the “Chart & Statistics” icon, the “Compare Subgroups” function was used, and for the visualization, the box plot was used. For the DNA methylation levels compression, beta values were used.

#### 2.2.3 Comparison of Metastatic Status and C1GalT1 Expression Levels

C1GalT1 gene expression levels between M0 and M1 status were compared by using the UCSC Xena portal. After selecting the corresponding study of TCGA for the cancer type, for the “Genomic” data type, the “C1GALT1” gene and the “Gene Expression” dataset were selected. For the “Phenotype” data type, the “sample_type” and the “pathologic_M” datasets were selected, which include data for tumor and normal tissue samples and M0 and M1 status, respectively. Only “primary tumor” tissues were selected to get rid of normal tissue samples, and samples with Mx status were removed as well. Later, samples with null values were removed. For the analysis in the “Chart & Statistics” icon “Compere Subgroups” function was used. For the visualization box plot was used. For the gene expression compressions, median gene expression values which were calculated as log2(normalized counts+1) were used.

#### 2.2.4 The Kaplan–Meier Plotter

Survival analysis was performed using the TCGA datasets, with Kaplan–Meier plots (KM) generated via the UCSC Xena portal. After selecting the corresponding study of TCGA for the cancer type, for the “Genomic” data type, the “C1GALT1” gene and the “Gene Expression” dataset were selected. For the “Phenotype” data type, “sample_type” was selected, which includes data for tumor and normal tissue samples. Only “primary tumor” tissues were selected; later, samples with null values were removed. Finally, the “Kaplan Meier Plot” function was used.

### 2.3 The TIMER2.0 Web Portal

The TIMER2.0 (http://timer.cistrome.org) web portal serves to explore tumor immunological, clinical, and genomic features comprehensively [14, 15].

#### 2.3.1 C1GALT1 Gene Expression Levels Validation

TIMER2.0 was used to validate the Xena portal results of the C1GALT1 gene expression levels in normal versus tumor tissues. In this study, the “Gene_DE” module was selected with the “C1GalT1” gene. Later, the Wilcoxon test computed the statistical significance for different types of tumor and normal tissue samples using TCGA data sets in the portal.

#### 2.3.2 The Relationship of the C1GALT1 Gene and C1GALT1C1 (Cosmc) Gene Expression Levels

To understand the relationship between C1GALT1 gene (T‐synthase) expression and C1GALT1C1 (Cosmc) gene expression levels in different tumor types, the TIMER2.0 web portal was used. In the portal, the “Gene_Corr” module was selected with the “C1GalT1” and “C1GalT1C1” genes with the “Purity Adjustment” function. Later, Spearman's rho values between two gene expression levels were calculated for different types of tumor samples by using the TCGA data sets in the portal.

#### 2.3.3 C1GALT1 Gene Relationship With Tumor Metastasis‐Related Genes

TIMER2.0 was used to understand the C1GALT1 gene expression relationship with genes that were found to have positive and negative relationships with metastasis. These genes were selected from previously published studies [16, 17]. In the TIMER2.0 web portal, the “Gene_Corr” module was selected with the “C1GalT1” and metastasis‐related gene types one by one with the “Purity Adjustment” function. Later, Spearman's rho values between two gene expression levels were calculated for different types of tumor samples by using the TCGA data sets in the portal.

#### 2.3.4 C1GALT1 Gene Expression Correlation With Tumor Proliferation

TIMER2.0 was used to investigate the correlation between C1GalT1 and proliferation‐related genes' expressions across various cancer types, aiming to understand the potential impact of C1GalT1 expression on tumor proliferation. These genes were selected from previously published studies [18, 19, 20, 21, 22, 23, 24, 25]. In the TIMER2.0 web portal, the “Gene_Corr” module was selected with the “C1GalT1” and related gene types one by one with the “Purity Adjustment” function. Later, Spearman's rho values between two gene expression levels were calculated for different types of tumor samples by using TCGA data sets in the portal.

### 2.4 Comparison of Tumor‐Infiltrating Tregs/MDSCs and C1GalT1 Expression Levels

For this, the TISIDB (http://cis.hku.hk/TISIDB/index.php) was used. This web portal focuses on tumor and immune system interactions, integrating various heterogeneous data types such as PubMed, high‐throughput screening data, exome and RNA sequencing data sets of patient cohorts, The Cancer Genome Atlas (TCGA), and other public databases [26]. This integrated platform was utilized to examine the association between the presence of tumor‐infiltrating lymphocytes (TILs) and the expression of the C1GALT1 gene.

The C1GALT1 gene was selected for the “Gene Symbol” in the TISIDB web portal, and the “Lymphocyte” section was browsed. Later, Spearman correlations between C1GalT1 expression levels and immune cells across different cancer types were calculated. To be able to analyze the relation between each cancer type and Tregs and MDSCs individually, the plot function was used.

### 2.5 Statistical Analysis

The differences in the C1GalT1 gene expression and the average gene DNA methylation levels between tumor and normal tissues, as well as between M1 and M0 statuses, were analyzed by Welch's t‐test, and *p* < 0.05 was considered statistically significant. The relationship between the C1GalT1 gene expression and overall survival (OS) was analyzed by the KM plot model. The survival rates between the two groups were compared by the Log‐Rank test. The test level was *α* = 0.05, and *p* < 0.05 was considered as statistically significant. For the correlation with the tumor proliferative index, Spearman's test was used. A positive correlation was defined by *p* < 0.05 and Spearman's rank correlation coefficient (Rho) greater than 0; a negative correlation was defined by *p* < 0.05 and Rho < 0. A *p*‐value greater than 0.05 was considered not significant.

## 3 Results

### 3.1 Comparison of the C1GalT1 Gene Expression Between Tumor Versus Normal Tissues

**FIGURE 1:**
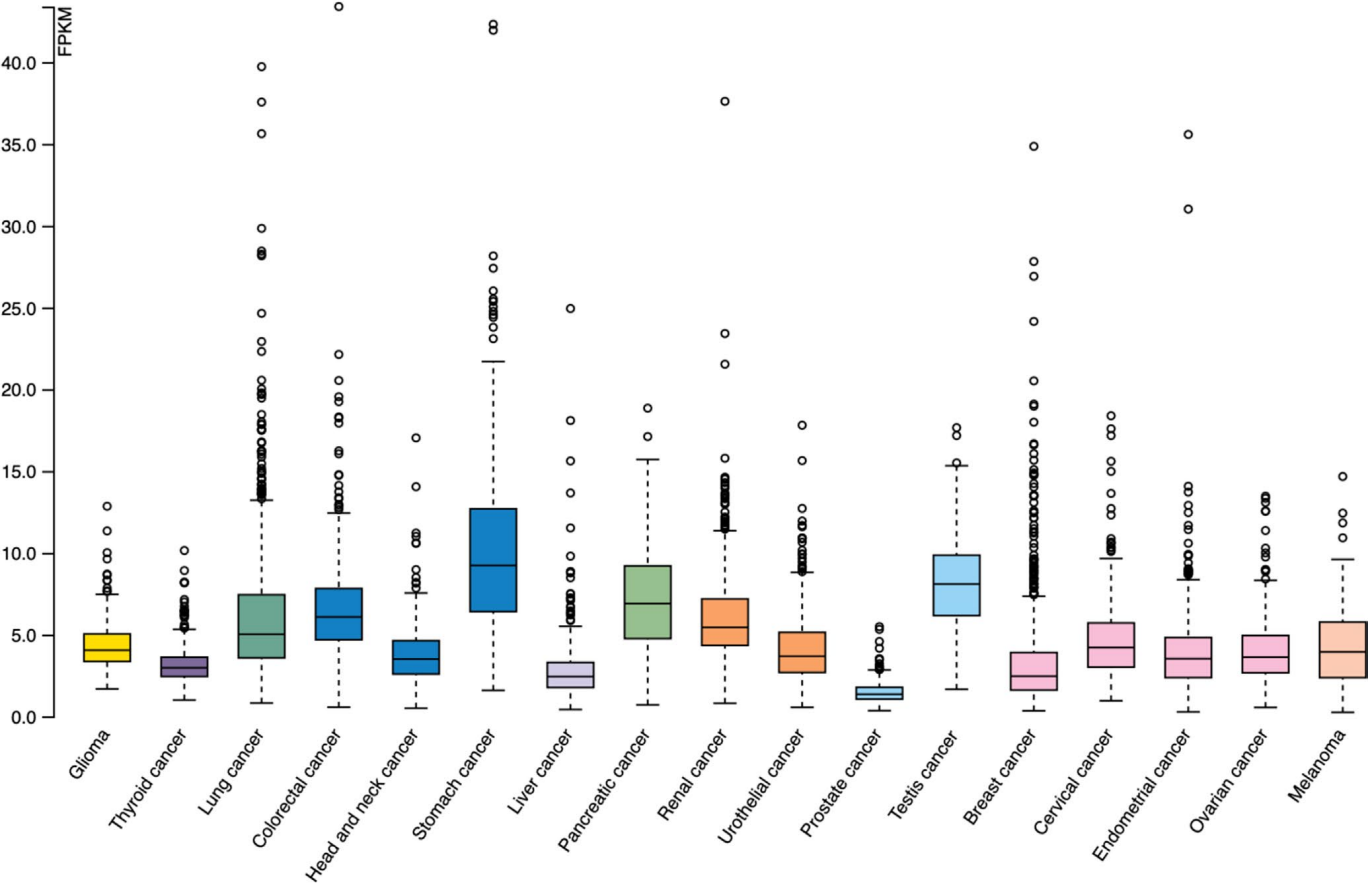
C1GalT1 gene expression differences between different cancer types.

The expression levels of the C1GalT1 gene (median FPKM) in different cancer types were compared using the HPA database. The highest C1GalT1 median FPKM was seen in stomach cancer, and testis, pancreatic, and colorectal cancers were followed, respectively. The lowest expressions were seen in prostate, liver, and breast cancer. The results are shown in Figure 1 and Table 1.

**TABLE 1:** C1GalT1 median gene expression levels between different cancer types.

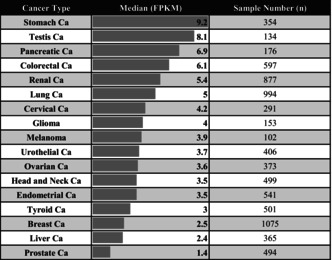

Abbreviations: Ca: cancer; FPKM: fragments per kilobase of transcript per million mapped reads.

The expression levels of the C1GalT1 gene in tumor and normal tissue were compared in log2 fold change using the Xena web portal and the TCGA cancer datasets. Tumor tissues in stomach, pancreatic, colorectal, lung, bladder, and endometrioid cancers exhibited significantly higher median C1GalT1 expression than healthy tissues.

For stomach cancer, normal tissues had a lower median of C1GALT1 gene expression level compared to tumor tissues (*p* = 0.00626 × 10^−3^). Pancreatic cancer also revealed substantial differences (*p* = 0.01981), with normal tissues having a lower median of C1GALT1 gene expression than tumor tissues. Colon cancer followed a similar trend, showing lower C1GALT1 levels in normal tissues compared to tumor tissues (*p* = 2.487e‐18). There were significant differences in lung cancer, with normal tissues having lower levels than tumor tissues (*p* = 3.537e‐22). Bladder cancer also demonstrated significant differences, with normal tissues having lower expression levels than the tumor ones (*p* = 0.0007669). Endometrioid‐type endometrial cancer also showed substantial differences, with tumor tissues having higher levels than normal tissues (*p* = 5.918e‐12).

**FIGURE 2:**
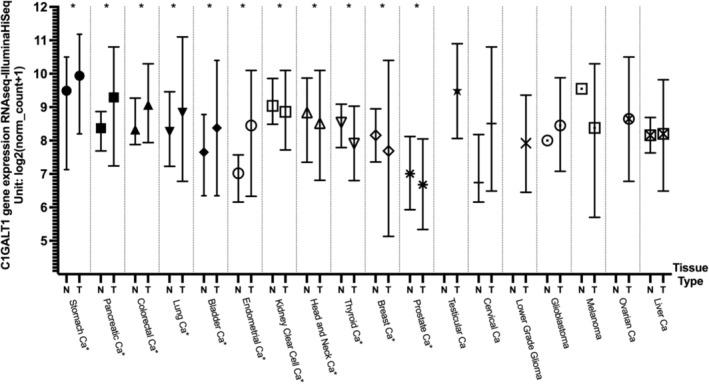
C1GalT1 gene expression differences between normal and tumor samples across cancer types on box‐plot. *Indicating statistically significant results. Ca: cancer; N: normal tissue; T: tumor tissue.

On the other hand, in kidney clear cell carcinoma, head and neck, thyroid, breast, and prostate cancers, normal tissue had higher expression of C1GALT1 than tumor tissues. For kidney clear cell carcinoma, normal tissues had a higher median expression of C1GALT1 compared to tumor tissues (*p* = 0.009444 × 10^−3^). In head and neck cancer, normal tissues exhibited significantly higher gene expression levels than tumor tissues (*p* = 0.01034). Breast cancer showed a similar trend, with normal tissues significantly exceeding C1GALT1 gene expression levels compared to tumor tissues (*p* = 4.876e‐13). Similarly, for prostate cancer, normal tissues displayed a higher median expression compared to tumor tissues (*p* = 0.006.49 × 10^−2^). In cervical cancer, glioblastoma, and liver cancer, the differences in C1GalT1 expression between normal and tumor tissues were not statistically significant. For glioma, testicular cancer, melanoma, and ovarian cancer, normal tissue samples were absent in the datasets; therefore, calculations could not be done. The results are shown in Figure 2 and summarized in Table 2.

**TABLE 2:** Median C1GalT1 gene expression differences between normal and tumor samples across different cancer types.

Cancer type	Tissue type	Median (log2(norm_count + 1))	Sample number (*n*)	Welch's *t*‐test (*p*)
Stomach Ca	Normal	9.49	35	0.000006264 (*t* = −5.242)
	Tumor	9.94	415	
Pancreatic Ca	Normal	8.37	4	0.01981 (*t* = −4.276)
	Tumor	9.29	178	
Colorectal Ca	Normal	8.33	51	2.487e‐18 (*t* = −11.57)
	Tumor	9.07	380	
Lung Ca	Normal	8.26	110	3.537e‐22 (*t* = −10.87)
	Tumor	8.83	1017	
Bladder Ca	Normal	7.65	19	0.0007669 (*t* = −3.953)
	Tumor	8.38	407	
Endometrioid Ca	Normal	7.02	24	5.918e‐12 (*t* = −10.01)
	Tumor	8.45	176	
Kidney clear cell Ca	Normal	9.04	72	0.000009444 (*t* = −4.037)
	Tumor	8.86	533	
Head and neck Ca	Normal	8.84	44	0.01034 (*t* = −2.657)
	Tumor	8.52	520	
Thyroid Ca	Normal	8.54	59	2.912e‐18 (*t* = −11.15)
	Tumor	7.91	505	
Breast Ca	Normal	8.16	114	4.876e‐13 (*t* = −7.541)
	Tumor	7.69	1097	
Prostate Ca	Normal	7.01	52	0.00006490 (*t* = −4.283)
	Tumor	6.68	497	
Testicular Ca	Normal	NA	NA	NA
	Tumor	9.49	150	
Cervical Ca	Normal	6.74	3	0.09324 (*t* = −3.002)
	Tumor	8.51	303	
Lower grade glioma	Normal	NA	NA	NA
	Tumor	7.92	516	
Glioblastoma	Normal	8	5	0.4218 (*t* = −0.8892)
	Tumor	8.45	154	
Melanoma	Normal	9.55	1	NA
	Tumor	8.38	103	
Ovarian Ca	Normal	NA	NA	NA
	Tumor	8.65	303	
Liver Ca	Normal	8.16	50	0.2147 (*t* = −1.248)
	Tumor	8.2	371	

Abbreviations: Ca: cancer; e: 10^; NA: not available.

Additionally, C1GALT1 gene expression levels were compared between normal and tumor tissues using the TIMER2.0 portal to validate the findings from the Xena portal. Consistent with the Xena results, tumor tissues of the stomach, pancreas, lung, and bladder cancers showed significantly higher C1GALT1 expression compared to normal tissues. While TIMER2.0 did not include a specific category for endometrioid cancer, it combined cervical and endometrial cancers; this group also demonstrated significantly elevated expression in tumor tissues, mirroring the Xena results. Similarly, instead of a combined colorectal cancer category, TIMER2.0 listed colon adenocarcinoma and rectal carcinoma separately. Both showed higher expression in tumor tissues, although the increase in rectal carcinoma was not statistically significant—again aligning with the Xena findings. Furthermore, kidney clear cell carcinoma, thyroid, breast, and prostate tumor tissues showed significantly lower C1GALT1 expression than normal tissues in both portals. The main discrepancy was observed in head and neck cancer, where TIMER2.0 showed similar expression levels between tumor and normal tissues, resulting in no statistically significant difference, unlike the findings in the Xena portal. The TIMER2.0 portal results are shown in Figure S1.

### 3.2 C1GalT1 DNA Methylation Levels Comparison Between Tumor Versus Normal Tissues

Average DNA methylation levels of the C1GalT1 gene were analyzed between tumor and normal tissues using beta values from the Xena web portal and TCGA datasets. Cancer types with significant differences in the C1GalT1 gene expression were examined to determine whether changes in DNA methylation could explain these variations.

In pancreatic cancer, the average DNA methylation level in normal tissues was significantly higher compared with tumor tissues (*p* = 0.0001507). Colorectal cancer exhibited a similar pattern, with normal tissues showing a higher average DNA methylation level than that observed in tumor tissues (*p* = 0.003155). The average DNA methylation level in normal tissues for bladder cancer was again higher than in the tumor (*p* = 0.03481). Endometrioid cancer also demonstrated a similar pattern, with normal tissue having a higher average DNA methylation level compared to tumor tissue (*p* = 0.000441).

For stomach cancer, the average DNA methylation level in normal tissues was higher than that observed in tumor tissues. However, the difference was not statistically significant (*p* = 0.922), likely due to the low sample size in normal tissues. Additionally, in lung cancer, normal tissues had a lower DNA methylation gene average than tumors, with the differences not being statistically significant as well (*p* = 0.315).

**FIGURE 3:**
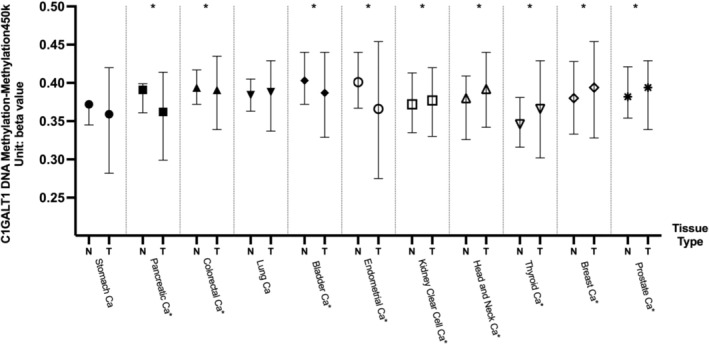
C1GalT1 DNA methylation level differences between normal and tumor samples across cancer types on box‐plot. *Indicating statistically significant results. Ca: cancer; N: normal tissue; T: tumor tissue.

**FIGURE 4:**
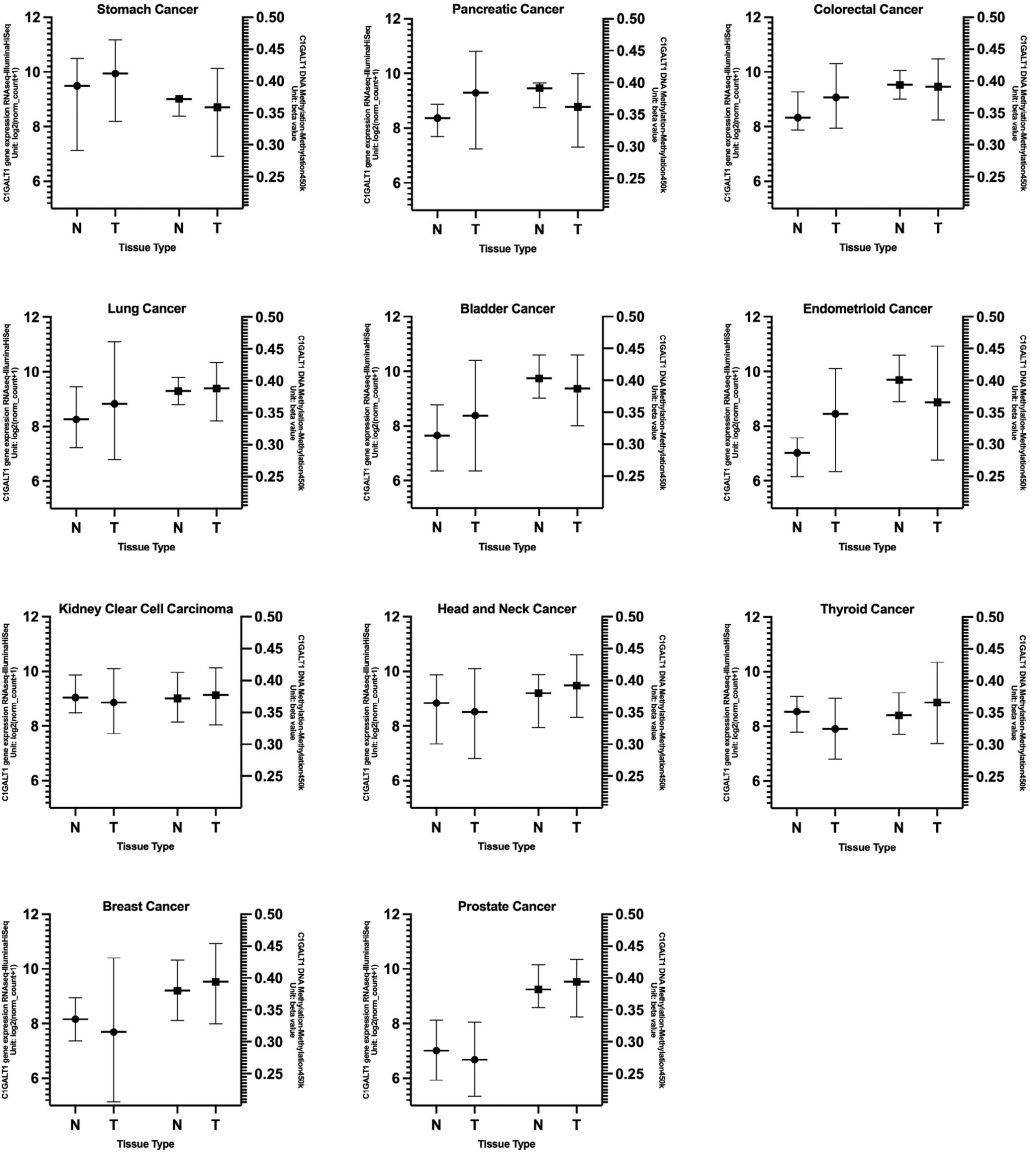
C1GALT1 gene expression between normal and tumor tissues, along with their corresponding DNA methylation data. N: normal tissue; T: tumor tissue.

In kidney clear cell carcinoma, tumor tissues exhibited higher average methylation levels than normal tissues (*p* = 0.03495). In head and neck cancer, tumor tissues again had higher average methylation levels compared to normal tissues (*p* = 0.00002215). Similarly, for thyroid cancer, normal tissues displayed a lower average DNA methylation level than tumor tissues (*p* = 0.001289 × 10^−6^). In breast cancer, normal tissues also showed a significantly lower average DNA methylation level compared to tumor tissues (*p* = 0.01436). Likewise, in prostate cancer, tumor tissues exhibited a higher average methylation level compared to normal tissues (*p* = 0.003637). These results may suggest that DNA methylation levels in tumor tissues could be one mechanism by which C1GALT1 is regulated. However, these results need to be further confirmed with in vivo, in vitro studies as well as checking other active and repressive histone methylation markers in further studies. The average DNA methylation levels across various tumor types are shown in Figure 3 and summarized in Table 3. Tumor types with statistically significant differences in C1GALT1 gene expression between normal and tumor tissues, along with their corresponding DNA methylation data, are presented side by side in Figure 4 to illustrate the correlation between expression levels and methylation status.

**TABLE 3:** Median C1GalT1 DNA methylation level differences between normal and tumor samples across different cancer types.

Cancer type	Tissue type	Median (beta value)	Sample number (*n*)	Welch's *t*‐test (*p*)
Stomach Ca	Normal	0.372	2	0.9222 (*t* = −0.1216)
	Tumor	0.359	396	
Pancreatic Ca	Normal	0.391	10	0.0001507 (*t* = −5.320)
	Tumor	0.362	184	
Colorectal Ca	Normal	0.394	45	0.003155 (*t* = −3.061)
	Tumor	0.391	395	
Lung Ca	Normal	0.384	75	0.3149 (*t* = −1.008)
	Tumor	0.388	830	
Bladder Ca	Normal	0.403	21	0.00002959 (*t* = −5.189)
	Tumor	0.387	410	
Endometrial Ca	Normal	0.401	46	2.427e‐18 (*t* = −11.41)
	Tumor	0.366	431	
Kidney clear cell Ca	Normal	0.372	160	0.03495 (*t* = −2.118)
	Tumor	0.377	319	
Head and neck Ca	Normal	0.38	50	0.00002215 (*t* = −4.608)
	Tumor	0.392	528	
Thyroid Ca	Normal	0.346	56	1.289e‐9 (*t* = −6.824)
	Tumor	0.366	506	
Breast Ca	Normal	0.38	98	0.01511 (*t* = −2.460)
	Tumor	0.394	785	
Prostate Ca	Normal	0.382	50	0.003637 (*t* = −3.016)
	Tumor	0.394	498	

Abbreviations: Ca: cancer; e: 10^.

### 3.3 The Relationship Between C1GalT1 and C1GalT1C1(Cosmc) Genes Expression Levels

The expression and function of T‐synthase (C1GALT1) depend on the presence of its molecular chaperone, Cosmc [6]. Therefore, understanding the relationship between the Cosmc gene and C1GALT1 is crucial. For this analysis, the TIMER2.0 web portal was used with Spearman's correlation. Statistically significant positive correlations between C1GALT1 and Cosmc expression levels were observed across all cancer types examined—including stomach, pancreas, colon, rectum, cervical/endocervical, bladder, kidney‐clear cell, head and neck, thyroid, breast, prostate, testicular, liver, glioblastoma, and melanoma—except for ovarian cancer. Although the correlation in ovarian cancer was positive, it did not reach statistical significance. The results are listed in Table 4.

**TABLE 4:** The relationship (Spearman's *p* correlation and rho values) between C1GALT1 and C1GALT1C1(Cosmc) gene in various cancer types.

Cancer types	Rho	*p*	Purity adjusted *p* values
Stomach cancer STAD (*n* = 415)	0.456	6.590948320285E‐21	5.272758656228E‐20
Pancreatic adenocarcinoma PAAD (*n* = 179)	0.482	2.49171910574108E‐11	5.24572443313912E‐11
Colon adenocarcinoma COAD (*n* = 458)	0.367	1.99826375710378E‐14	5.91556590455316E‐14
Rectum adenocarcinoma READ (*n* = 166)	0.462	1.01156476838433E‐08	1.83920866978969E‐08
Cervical and endocervical cancer CESC (*n* = 306)	0.358	8.65480268530531E‐10	1.64853384482006E‐09
Bladder urothelial carcinoma BLCA (*n* = 408)	0.196	4.56658688495229E‐10	9.13317376990459E‐10
Kidney clear cell carcinoma KIRC (*n* = 533)	0.480	5.83067550983456E‐28	5.83067550983456E‐27
Head and neck cancer HNSC (*n* = 522)	0.192	1.71417097721549E‐05	2.85695162869248E‐05
Thyroid cancer THCA (*n* = 509)	0.568	5.50795406247475E‐43	2.2031816249899E‐41
Breast cancer BRCA (*n* = 1100)	0.196	4.56658688495229E‐10	9.13317376990459E‐10
Prostate cancer PRAD (*n* = 498)	0.433	1.88651314565863E‐20	1.07800751180493E‐19
Testicular germ cell tumors TGCT (*n* = 150)	0.249	0.00239394267490474	0.00294777925808182
Glioblastoma GBM (*n* = 153)	0.330	8.16860485639619E‐05	0.000125670843944557
Melanoma SKCM (*n* = 471)	0.493	2.67619584141664E‐29	5.35239168283328E‐28
Ovarian cancer OV (*n* = 303)	0.119	0.060770479375403	0.063968925658319
Liver hepatocellular carcinoma LIHC (*n* = 371)	0.415	8.56598316569245E‐16	3.80710362919664E‐15

*Note:* 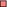 Spearman's *p* positive correlation (*p* < 0.05, *p* > 0); 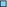 Spearman's *p* negative correlation (*p* < 0.05, *p* < 0); 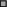 Not significant (*p* > 0.05).

### 3.4 Metastatic Status and G1GalT1 Expression Level

**FIGURE 5:**
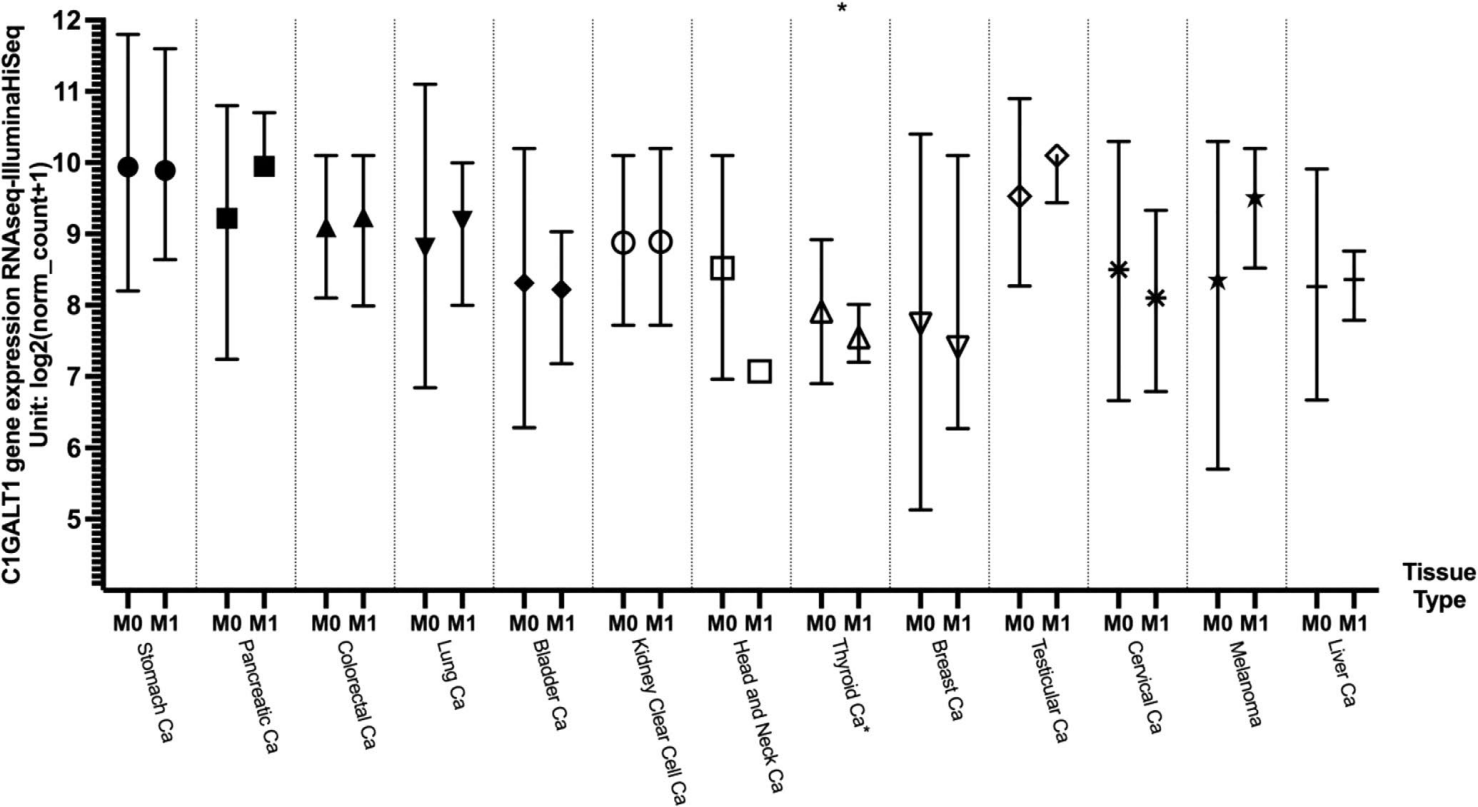
C1GalT1 gene expression differences between metastatic (M1) and non‐metastatic (M0) tissues across different cancer types. *Indicating statistically significant results. Ca: cancer; M0: non‐metastatic tissue; M1: metastatic tissue.

The C1GalT1 mean RNA expression levels based on metastatic status were compared in log2 fold change using the Xena web portal using the TCGA cancer datasets. Unfortunately, most of the results were not statistically significant due to the low sample size in the M1 subgroups. Thyroid cancer was the only cancer type with a statistically significant difference between M0 versus M1 samples, with the M0 subgroup having higher mean C1GalT1 gene expression (7.91; *n* = 282) compared to the M1 subgroup (7.54; *n* = 8; *p* = 0.007837). The results are shown in Figure 5 and summarized in Table 5.

**TABLE 5:** Median C1GalT1 gene expression differences between metastatic (M1) and non‐metastatic (M0) tissues across different cancer types.

Cancer type	Metastasis	Median (log2(norm_count + 1))	Sample number (*n*)	Welch's *t*‐test (*p*)
Stomach Ca	M0	9.94	364	0.2334 (*t* = −1.217)
	M1	9.89	27	
Pancreatic Ca	M0	9.22	80	0.3303 (*t* = −1.147)
	M1	9.95	4	
Colorectal Ca	M0	9.1	255	0.5197 (*t* = 0.6487)
	M1	9.24	38	
Lung Ca	M0	8.8	757	0.4137 (*t* = −0.8319)
	M1	9.18	23	
Bladder Ca	M0	8.31	196	0.6974 (*t* = −0.3984)
	M1	8.22	11	
Endometrial Ca	M0	NA	NA	NA
	M1			
Kidney clear cell Ca	M0	8.88	422	0.9342 (*t* = −0.08279)
	M1	8.89	79	
Head and neck Ca	M0	8.52	186	NA
	M1	7.07	1	
Thyroid Ca	M0	7.91	282	0.007837 (*t* = −3.470)
	M1	7.55	8	
Breast Ca	M0	7.74	904	0.8872 (*t* = −0.1435)
	M1	7.41	22	
Prostate Ca	M0	NA	NA	NA
	M1			
Testicular Ca	M0	9.53	115	0.4035 (*t* = −1.251)
	M1	10.1	2	
Cervical Ca	M0	8.5	115	0.1623 (*t* = −1.521)
	M1	8.1	9	
Lower grade glioma	M0	NA	NA	NA
	M1			
Glioblastoma	M0	NA	NA	NA
	M1			
Melanoma	M0	8.35	98	0.09712 (*f* = 2.165)
	M1	M1a:8.52, M1b:10.2, M1c:9.81	3	
Ovarian Ca	M0	NA	NA	NA
	M1			
Liver Ca	M0	8.26	266	0.6358 (*t* = 0.5198)
	M1	8.36	4	

Abbreviations: Ca: cancer; e:10^; M0: non‐metastatic; M1: metastatic; NA: not available.

### 3.5 C1GALT1 Gene Relationship With Tumor Metastasis‐Related Genes

To further understand the relationship between C1GALT1 gene expression and metastasis, C1GALT1 gene expression was analyzed with the genes that were found to have positive and negative relationships with metastasis using the TIMER2.0 portal. These genes were selected from previously published studies [16, 17], and Spearman's *p* correlation method was used to analyze the relationship.

First, the genes that showed metastatic enrichment across multiple cancers (TP53, CDKN2A, and TERT) and genes that had a significant frequency bias in at least one metastatic cancer type (Amplification/Deletion/Mutation: AR‐prostate cancer; CCND1 and RSF1‐skin melanoma; CREBBP and TSC2‐pancreatic neuroendocrine tumor; ESR1‐breast cancer; PTEN‐prostate cancer; PTPRD‐kidney clear cell carcinoma, and RET‐thyroid) were selected [16].

The expression levels of the RSF1 gene were positively correlated with C1GALT1 gene expression levels in all cancer types, including melanoma. Similarly, the CREBBP gene was positively correlated with C1GALT1 gene expression levels in all cancer types, including pancreatic cancer, except stomach cancer.

TP53 was positively correlated with C1GALT1 in prostate, liver, and glioblastoma, but negatively in colon cancer. CDKN2A showed a positive correlation in stomach, bladder, prostate, and liver; negative in rectal, cervical, kidney‐clear cell, thyroid, and testicular cancers. TERT was positively correlated only in testicular and liver cancers, and negatively in rectal cancer.

The AR gene showed a positive correlation with C1GALT1 expression not only in prostate cancer but also in other cancer types, including pancreatic, rectal, kidney‐clear cell, head and neck, thyroid, testicular, glioblastoma, melanoma, and liver cancers. The TSC2 gene exhibited a positive correlation with C1GALT1 expression in pancreatic and liver cancers, while showing a negative correlation in colon, rectal, kidney‐clear cell, and melanoma cancers. Similarly, PTEN was positively correlated with C1GALT1 expression in prostate cancer as well as in pancreatic, colon, rectal, cervical/endocervical, bladder, kidney‐clear cell, head and neck, thyroid, breast, liver cancers, and melanoma. The PTPRD gene was positively correlated with C1GALT1 expression in kidney‐clear cell carcinoma and other cancers such as colon, head and neck, thyroid, prostate, breast, ovarian, liver cancers, and melanoma.

CCND1 showed no correlation with C1GALT1 in melanoma but was positively correlated in several other cancers. ESR1 was not correlated in breast cancer but showed positive associations in rectal, bladder, head and neck, prostate, kidney‐clear cell, glioblastoma, and melanoma. RET was not correlated in thyroid cancer but was positively associated in prostate and head and neck cancers. The results are shown in Table 6.

**TABLE 6:** The correlation between C1GALT1 gene expression and tumor metastasis‐related genes in various cancer types.

Cancer types	AR	CCND1	CDKN2A	CREBBP	ESR1	PTEN	PTPRD	RET	RSF1	TERT	TP53	TSC2
Stomach cancer STAD (*n* = 415)	−0.09	0.264	0.168	0.028	−0.08	0.072	−0.03	−0.073	0.174	0.08	−0.054	0.021
Pancreatic adenocarcinoma PAAD (*n* = 179)	0.175	0.452	0.059	0.339	−0.058	0.185	0.002	−0.115	0.479	0.147	0.063	0.201
Colon adenocarcinoma COAD (*n* = 458)	0.092	0.305	−0.054	0.214	0.031	0.323	0.136	−0.043	0.488	−0.091	−0.144	−0.193
Rectum adenocarcinoma READ (*n* = 166)	0.182	0.313	−0.176	0.323	0.252	0.483	0.063	−0.074	0.64	−0.198	0.004	−0.308
Cervical and endocervical cancer CESC (*n* = 306)	0.089	0.079	−0.144	0.23	0.117	0.186	0.043	−0.102	0.39	0.007	−0.059	−0.026
Bladder urothelial carcinoma BLCA (*n* = 408)	0.067	−0.027	0.294	0.266	0.205	0.217	0.088	0.028	0.406	0.07	−0.087	−0.016
Kidney clear cell carcinoma KIRC (*n* = 533)	0.479	0.29	−0.105	0.369	0.243	0.368	0.197	−0.074	0.482	−0.05	−0.087	−0.102
Head and neck cancer HNSC (*n* = 522)	0.228	0.298	−0.032	0.28	0.183	0.347	0.106	0.263	0.428	−0.042	0.031	−0.002
Thyroid cancer THCA (*n* = 509)	0.513	0.24	−0.161	0.532	0.081	0.544	0.152	0.015	0.606	−0.035	0.019	0.011
Breast cancer BRCA (*n* = 1100)	−0.019	−0.098	0.057	0.159	−0.166	0.225	0.183	−0.079	0.334	0.01	−0.025	−0.252
Prostate cancer PRAD (*n* = 498)	0.571	0.309	0.119	0.574	0.312	0.16	0.263	0.138	0.674	−0.093	0.106	0.076
Testicular germ cell tumors TGCT (*n* = 150)	0.162	−0.192	−0.205	0.295	−0.157	0.051	−0.05	0.321	0.345	0.358	0.029	−0.11
Glioblastoma GBM (*n* = 153)	0.397	−0.181	0.085	0.267	0.222	0.148	0.106	−0.1	0.3	0.048	0.182	−0.068
Melanoma SKCM (*n* = 471)	0.255	0.055	−0.003	0.251	0.36	0.287	0.171	−0.043	0.441	0.088	−0.048	−0.143
Ovarian cancer OV (*n* = 303)	0.059	0.144	0.017	0.148	0.098	0.098	0.132	−0.023	0.247	0.087	0.12	0.092
Liver hepatocellular carcinoma LIHC (*n* = 371)	0.148	0.261	0.189	0.539	−0.07	0.435	0.148	0.079	0.613	0.158	0.267	0.175

*Note:* 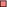 Spearman's *p* positive correlation (*p* < 0.05, *p* > 0); 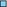 Spearman's *p* negative correlation (*p* < 0.05, *p* < 0); 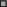 Not significant (*p* > 0.05).

**FIGURE 6:**
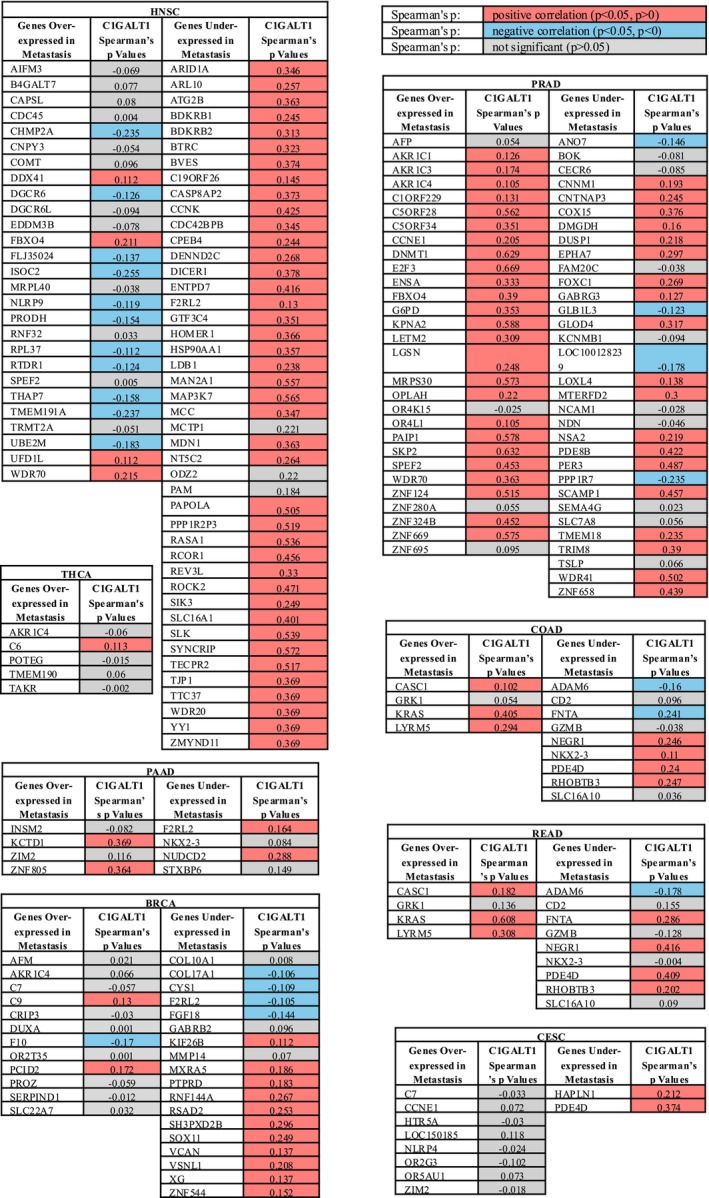
The relationship between C1GALT1 gene expression and genes that were over/under‐expressed in metastasis.

Moreover, other genes that were over‐ or under‐expressed in metastasis of specific cancer types [17] were also analyzed by using the TIMER2.0 portal. Except for prostate and head and neck cancers, some genes were positively correlated, and some genes were negatively correlated with C1GALT1 in metastasis. In prostate cancer, most of the genes that were overexpressed in metastasis of prostate cancer were mainly positively associated with C1GALT1. In addition, in head and neck cancer, the C1GALT1 gene expression was mostly negatively correlated with over‐expressed genes in metastasis and positively correlated with under‐expressed genes in metastasis. The results are shown in Figure 6.

### 3.6 Prognostic Analysis of C1GalT1 Gene

**FIGURE 7:**
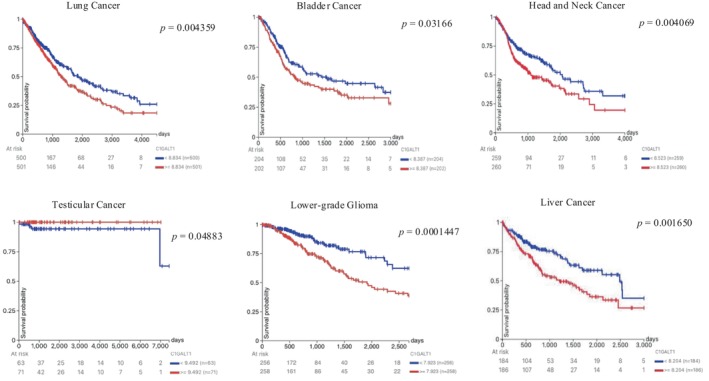
Overall survival (OS) comparison between high and low C1GalT1 gene expression groups displayed on KM plots across different cancer types.

The overall survival associated with the C1GalT1 gene in various cancer types was analyzed using the Xena web portal with KM plots. Statistically significant differences in overall survival (OS) were observed between low and high C1GalT1 expression groups in lung, bladder, head and neck, testicular and liver cancers, and glioma/glioblastoma. In lung, bladder, head and neck, and liver cancers, the low C1GalT1 expression group demonstrated statistically significantly better OS than the higher expression group. Similarly, in lower‐grade glioma, the low C1GalT1 expression group exhibited a markedly greater OS compared to the high expression group. On the other hand, in testicular cancer, the high C1GalT1 expression group showed better OS compared to the low expression group. All statistically significant KM plots are shown in Figure 7. Statistically non‐significant ones are shown in Figure S2.

### 3.7 The C1GalT1 Gene Expression and Tumor Proliferation

To investigate the influence of C1GalT1 gene expression on tumor proliferation, the expression levels of C1GalT1 were compared with the genes that were previously associated with tumor proliferation by using the TIMER2.0 portal, and Spearman's *ρ* correlation method was used to analyze the relationship. The selected genes are the MKI67 gene (responsible for transcription of the Ki67 protein) [24, 25], PCNA gene (responsible for transcription of the PCNA protein) [24], MCM genes (responsible for transcription of the MCM2, 3, 4, 5, 6, and 7 proteins) [23, 24], MAP17 (PDZK1IP1) gene (responsible for transcription of the MAP17 protein) [21, 22], and PLK1 gene (responsible for transcription of the Polo‐like kinase) [18, 19, 20].

A significant positive correlation was observed between C1GALT1 and MKI67 expression in nearly all cancer types—except glioblastoma—including stomach, pancreas, colon, rectum, cervical, bladder, kidney‐clear cell, head and neck (HPV+/−), thyroid, breast, prostate, testicular, ovarian, liver, and melanoma. MCM6 showed similar correlations, except in HPV+ head and neck cancer, while MCM4 was positively correlated with C1GALT1 across all cancer types.

C1GALT1 expression was significantly positively correlated with all proliferation‐related genes in pancreatic, bladder, and breast cancers. In stomach, prostate, liver, and HPV− head and neck cancers, most genes were correlated except MCM5 or MAP17. In kidney‐clear cell carcinoma, all except MAP17 and PLK1 showed significant correlations. These results may suggest that C1GALT1 might be related to enhancing tumor proliferation in most cancer types. However, these results need to be validated further in in vivo and in vitro studies. The results are presented in Table 7.

**TABLE 7:** The correlation between the C1GalT1 gene expression and proliferation‐related genes in various cancer types.

Cancer types	MCM2	MCM3	MCM4	MCM5	MCM6	MCM7	MKI67	PCNA	MAP17 (PDZK1IP1)	PLK1
Stomach cancer STAD (*n* = 415)	0.134	0.231	0.166	−0.006	0.242	0.11	0.303	0.195	0.225	0.12
Pancreatic adenocarcinoma PAAD (*n* = 179)	0.302	0.43	0.36	0.162	0.267	0.259	0.404	0.342	0.241	0.31
Colon adenocarcinoma COAD (*n* = 458)	0.003	0.181	0.202	−0.165	0.282	0.071	0.289	0.14	−0.03	−0.042
Rectum adenocarcinoma READ (*n* = 166)	−0.027	0.362	0.3	−0.207	0.411	0.105	0.478	0.194	−0.168	−0.001
Cervical and endocervical cancer CESC (*n* = 306)	−0.127	0.227	0.205	−0.084	0.23	−0.175	0.186	−0.052	0.185	0.077
Bladder urothelial carcinoma BLCA (*n* = 408)	0.355	0.301	0.427	0.252	0.405	0.188	0.403	0.389	0.303	0.36
Kidney clear cell carcinoma KIRC (*n* = 533)	0.212	0.201	0.439	0.076	0.436	0.168	0.243	0.42	0.035	0.037
Head and neck cancer HNSC (*n* = 522)	0.1	0.099	0.319	0.072	0.16	0.051	0.31	0.054	−0.071	0.266
HNSC‐HPV− (*n* = 422)	0.138	0.155	0.316	0.128	0.204	0.102	0.336	0.124	−0.088	0.272
HNSC‐HPV+ (*n* = 98)	0.095	−0.002	0.382	0.067	0.156	−0.214	0.266	−0.191	−0.02	0.273
Thyroid cancer THCA (*n* = 509)	0.169	0.229	0.262	0.045	0.191	0.082	0.154	0.201	−0.107	0.118
Breast cancer BRCA (*n* = 1100)	0.189	0.193	0.279	0.184	0.347	0.112	0.315	0.181	0.17	0.206
Prostate cancer PRAD (*n* = 498)	0.526	0.543	0.646	0.284	0.576	0.123	0.488	0.54	0.03	0.376
Testicular germ cell tumors TGCT (*n* = 150)	0.393	0.336	0.552	0.413	0.47	0.458	0.284	0.335	−0.368	0.346
Glioblastoma GBM (*n* = 153)	0.19	0.305	0.256	0.006	0.259	0.167	0.093	0.187	0.197	−0.069
Melanoma SKCM (*n* = 471)	0.045	0.232	0.258	−0.069	0.31	0.066	0.194	0.381	−0.101	−0.025
Ovarian cancer OV (*n* = 303)	0.027	−0.035	0.169	0.041	0.199	0.037	0.189	0.022	−0.077	0.135
Liver hepatocellular carcinoma LIHC (*n* = 371)	0.513	0.534	0.554	0.437	0.573	0.505	0.502	0.494	−0.096	0.467

*Note:* 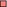
Spearman's *p* positive correlation (*p* < 0.05, *p* > 0); 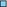 Spearman's *p* negative correlation (*p* < 0.05, *p* < 0); 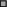 Not significant (*p* > 0.05).

### 3.8 Regulatory T‐Cells, MDSCs, and C1GalT1 Expression Levels

**FIGURE 8:**
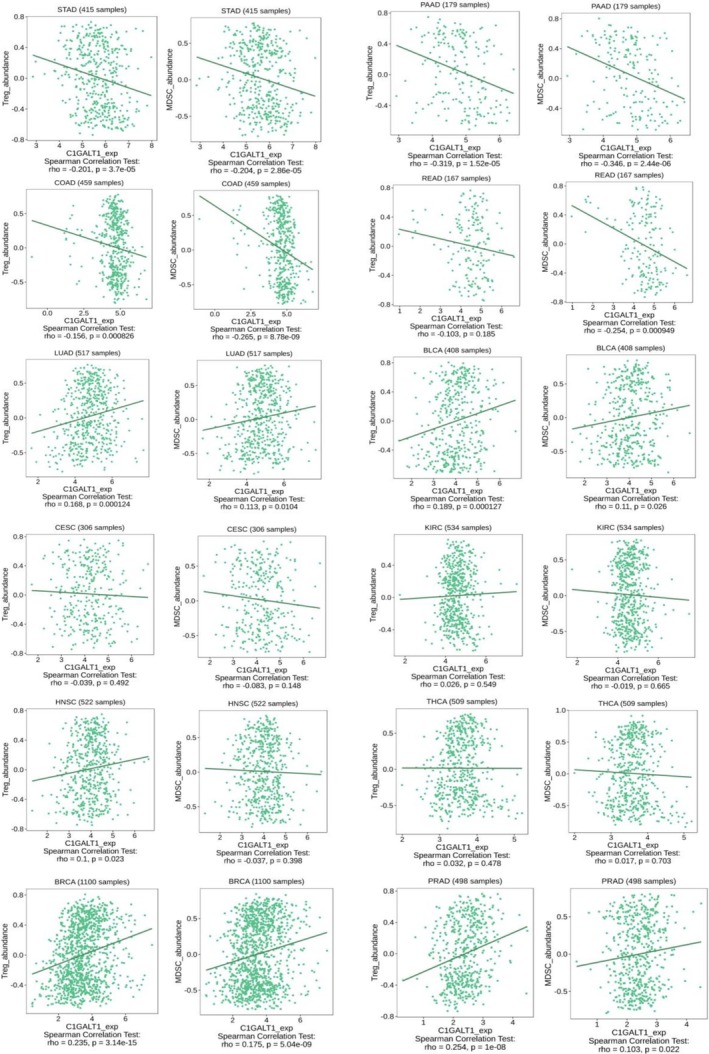
Tregs/MDSCs correlations with C1GalT1 expression levels in selected cancer types. Cancer types: BLCA: bladder urothelial carcinoma, BRCA: breast invasive carcinoma, CESC: cervical and endocervical cancers, COAD: colon adenocarcinoma, HNSC: head and neck squamous cell carcinoma, KIRC: kidney renal clear cell carcinoma, LUAD: lung adenocarcinoma, PAAD: pancreatic adenocarcinoma, PRAD: prostate adenocarcinoma, READ: rectum adenocarcinoma, STAD: stomach adenocarcinoma.

The study assessed the correlation between the C1GALT1 gene expression and the abundance of tumor‐infiltrating immunosuppressive Tregs and MDSCs across multiple cancer types. The distinct patterns of positive and negative correlations were observed across different cancer types. The correlations between C1GALT1 expression and both Tregs and MDSCs within the same cancer types were found to be parallel across all analyzed cancer types. A clear pattern of negative correlations was found in all the analyzed gastrointestinal cancer types, which were stomach adenocarcinoma, pancreatic adenocarcinoma, colon adenocarcinoma, and rectum adenocarcinoma. This negative correlation suggests that higher levels of C1GALT1 expression are associated with lower levels of both Tregs and MDSCs, which may point to a potential link between C1GALT1 expression and reduced immunosuppressive cell presence within the tumor microenvironment. However, this observation is based solely on statistical associations and requires further validation through in vivo and in vitro studies. A negative correlation was also found for cervical squamous cell carcinoma and endocervical adenocarcinoma, but it was statistically insignificant. Conversely, positive correlations were found in lung adenocarcinoma, bladder urothelial carcinoma, breast invasive carcinoma, and prostate adenocarcinoma. In these cases, increased C1GALT1 expression correlated with a higher abundance of Tregs and MDSCs, suggesting a potential immunosuppressive role for C1GALT1 that could facilitate tumor evasion from immune surveillance. Kidney renal clear cell carcinoma, head and neck squamous cell carcinoma, and thyroid carcinoma showed statistically insignificant correlations. This contrasting behavior underscores the complex and variable impact of C1GALT1 on the immune microenvironment across different cancer types. C1GalT1 expression and Tregs/MDSCs correlations in selected cancer types are shown in Figure 8.

## 4 Discussion

This study comprehensively analyzed the expression patterns, clinical correlations, and potential biological significance of the C1GALT1 gene across a wide range of cancer types using publicly available datasets and bioinformatics platforms. By integrating data from the Human Protein Atlas, UCSC Xena, TIMER2.0, and TISIDB, we systematically explored the relationships between C1GALT1 expression and tumor characteristics, including differential expression between tumor and normal tissues, DNA methylation status, associations with survival, tumor proliferation markers, metastatic gene signatures, and immune cell infiltration. While our findings consistently revealed statistically significant correlations between C1GALT1 and several oncogenic or immunoregulatory parameters, it is important to emphasize that these observations are based solely on association analyses. As such, they should not be interpreted as evidence of causality. Further functional validation using in vitro and in vivo models will be essential to confirm whether C1GALT1 plays a direct mechanistic role in tumor progression, immune modulation, or metastasis. Additionally, variations observed across different datasets highlight the need for context‐specific interpretations and underscore the biological complexity underlying C1GALT1‐mediated processes.

In colorectal cancer, overexpression of the T‐synthase enzyme has been reported [27, 28] and is associated with the production of TACAs, metastasis, and poor prognosis [29], findings consistent with our results. Similarly, in gastric cancer, the C1GalT1 gene and protein were significantly upregulated in tumor samples compared to healthy tissues, correlating with advanced disease stages and poor prognosis, consistent with our findings [30]. Additionally, another study demonstrated that C1GALT1 was overexpressed in 85% of pancreatic ductal adenocarcinoma tumors compared to adjacent non‐tumor tissues and was associated with poor survival outcomes in these patients. This observation also supports our results, showing high C1GalT1 levels in different tumor tissues [9]. Furthermore, consistent with our observations, studies have shown that C1GALT1 is overexpressed in lung adenocarcinoma tissues, with elevated expression levels strongly linked to poor prognosis in these patients [31]. Higher C1GALT1 expression was also seen in bladder, lung, and endometrial cancer types, which were similar to those in our study [32].

However, in previous studies, higher C1GALT1 expression was seen in breast, prostate, ovarian, and liver cancers [33], opposite to our study. Our analysis was transcriptomic analysis, and all breast cancer subtypes were grouped together for statistical evaluation, which might have obscured subtype‐specific variations in C1GALT1 expression. Also, these differences may reflect variations in tumor subtypes across studies, as well as biological differences between in silico, in vitro/in vivo models, and clinical samples. They also highlight the need for further research to better understand the context‐dependent effects of C1GALT1.

Moreover, in our study, cancers such as pancreatic, colorectal, bladder, and endometrioid demonstrated significantly higher DNA methylation levels in normal tissues compared to tumor tissues. These same cancers also exhibited elevated C1GALT1 expression in tumor tissues, suggesting that hypomethylation may contribute to increased gene expression—consistent with the general understanding that DNA methylation typically represses transcription. Conversely, in kidney‐clear cell, head and neck, thyroid, breast, and prostate cancers, tumor tissues showed significantly higher methylation levels than normal tissues. These cancers also displayed lower C1GALT1 expression in tumors, supporting the hypothesis that hypermethylation may suppress gene expression. Together, these correlative patterns between methylation status and gene expression across various tumor types suggest that DNA methylation may play a regulatory role in C1GALT1 expression in cancer. However, this association remains correlative rather than causal, and further validation through in vivo and in vitro studies—as well as investigations of other activating and repressive histone methylation markers—is necessary.

The strong positive correlation observed between C1GALT1 and Cosmc (C1GALT1C1) expression across multiple cancer types reinforces the well‐established functional dependency of T‐synthase on its specific molecular chaperone, Cosmc. Cosmc is required for proper folding and enzymatic activity of C1GALT1 [6]. Disruption in Cosmc expression or function has been shown to lead to misfolded or inactive C1GALT1 protein, resulting in the expression of truncated glycan structures such as the Tn antigen, which is frequently observed in various cancers and is associated with tumor progression and immune evasion [1]. Similar to our findings, increased C1GALT1(T‐synthase) and Cosmc expression levels were seen in an in vivo colorectal cancer study [8]. Additionally, it was shown that basal transcription of both Cosmc and T‐synthase is regulated by members of the specificity protein/Krüppel‐like factor (Sp/KLF) family, accounting for their widespread and coordinated expression patterns [34], which may explain our findings.

In head and neck squamous cell carcinoma, overexpression of C1GalT1 has been linked to poor prognosis and increased cell invasiveness. C1GalT1 modulates O‐glycosylation of the epidermal growth factor receptor, enhancing ligand‐binding affinity and downstream signaling, which promotes tumor progression. These findings align with our study and suggest that targeting C1GalT1 could offer therapeutic potential in HNSCC [35]. Additionally, in glioblastoma, knockdown models showed that C1GALT1 enhances the progression of glioma by regulating the O‐glycosylation and phosphorylation of EGFR and the subsequent downstream AKT/ERK signaling pathway [32], which was similar to our survival results showing that high C1GalT1 expression causes decreased OS. Furthermore, an in vivo study using cell lines and mouse models revealed that overexpression of C1GALT1 in hepatocellular carcinoma tissues was linked to advanced tumor stages, metastasis, and poor prognosis [36], findings that are parallel to those in our study. Collectively, these results underscore the critical role of C1GALT1 in cancer progression across multiple tumor types and its potential as a target for therapeutic intervention.

Previous studies have reported that altered glycosylation patterns, including truncated O‐glycans generated by aberrant C1GALT1 activity, can influence tumor progression, invasion, and immune evasion [37]. While this study identified significant differences in C1GALT1 expression levels between metastatic and non‐metastatic cancers only in thyroid cancer, these findings may be constrained by limited sample sizes in the M1 subgroups for other cancer types. Therefore, to further investigate the potential link between C1GALT1 and metastasis, we analyzed its expression in relation to metastasis‐associated genes. Previously, C1GALT1 upregulation was associated with tumor progression and metastasis in breast [38, 39], prostate [40], lung [40], liver [41], bladder [42], pancreas [43], stomach [44], and colon [45] cancers. In our study, most of the metastasis‐associated genes showed a positive correlation with C1GALT1 in prostate, breast, pancreas, bladder, and liver cancers, which was correlated with the previous studies. For colon and rectal cancer types, some metastasis‐associated genes were positively correlated, and some were negatively correlated with C1GALT1 in our study. Additionally, kidney‐clear cell carcinoma and melanoma cancer types showed a positive correlation in our study.

Our findings reveal a consistent and statistically significant positive correlation between C1GALT1 expression and key tumor proliferation markers across a wide range of cancer types, suggesting a potential role for C1GALT1 in promoting tumor cell proliferation. Among the proliferation‐associated genes analyzed—including MKI67, PCNA, MCM2–7, MAP17 (PDZK1IP1), and PLK1—many demonstrated strong positive correlations with C1GALT1 expression in cancers such as pancreas, bladder, breast, stomach, prostate, and liver. For instance, the MKI67 gene, a well‐established marker of cellular proliferation used widely in cancer grading and prognosis [24, 25], showed significant correlation with C1GALT1 in nearly all tumor types evaluated, except for glioblastoma. Similarly, MCM proteins, which are essential for DNA replication licensing and serve as emerging proliferation markers [23, 24], were significantly associated with C1GALT1 expression—most notably MCM4 and MCM6, which were consistently correlated across cancers. These results align with prior evidence suggesting that C1GALT1 can influence tumor behavior by enhancing cancer progression [46].

One study reported that C1GALT1 expression was notably higher in breast cancer tumors with high Ki67 levels compared to those with low Ki67 levels [47]. Similarly, both in vivo and in vitro studies demonstrated that hepatocellular carcinoma cells lacking C1GalT1 exhibited reduced Ki‐67 staining compared to wild‐type [36], and breast cancer tissues in C1GalT1‐deficient mice showed fewer Ki67‐positive cells [48]. In alignment with these findings, our study revealed a consistent positive correlation between CaGalT1 expression levels and MKI67, the gene encoding the Ki‐67 protein, across almost all examined cancer types. However, another study reported varying correlations between Ki‐67 levels and C1GalT1 expression across different breast cancer cell types in knockout experiments [39]. Additionally, an in vivo study suggested that the absence of C1GalT1 in pancreatic adenocarcinoma is linked with increased Ki‐67 expression [43]. The strongest associations—where C1GALT1 expression correlated with all tested proliferation markers—were found in pancreatic, bladder, and breast cancers, suggesting that C1GALT1 may play a particularly influential role in regulating proliferative capacity in these tumors. Overall, these findings support a hypothesis that C1GALT1 may contribute to enhanced tumor proliferation through transcriptional co‐regulation or downstream glycosylation‐dependent signaling mechanisms. However, these correlations remain associative, and further, in vitro and in vivo, functional studies are required to establish causality and define the mechanistic underpinnings of C1GALT1's role in cell cycle regulation, tumor growth, and metastasis.

The studies showed that C1GalT1 expression in tumor cells plays a dual role in modulating both tumor cell–cell interactions and tumor–macrophage communication through galectin‐3 and MGL, collectively influencing cancer development and progression. MGL was mainly found on macrophages and dendritic cells. Also, MGL is capable of binding to CD45 on effector T cells, leading to reduced proliferation and suppression of T cell‐mediated anti‐tumor responses [46, 49, 50, 51]. MGL showed an immunomodulator effect within the tumor microenvironment, interfering with Treg functions [52] and MDSC [53]. Our analysis of C1GalT1 expression with Tregs and MDSCs uncovered complex patterns. While a negative correlation was predominantly observed in gastrointestinal cancers, suggesting a possible immunosuppressive role for C1GalT1, other cancers like lung adenocarcinoma and breast invasive carcinoma showed positive correlations. These findings imply that C1GalT1 may contribute to immune evasion by enhancing the presence of immunosuppressive cells in the tumor microenvironment, which can vary significantly across different cancer types and reveal a complex pattern of interactions within the tumor microenvironment.

## 5 Limitations

This study provides a comprehensive pan‐cancer bioinformatic analysis of C1GALT1 expression, DNA methylation, immune interactions, and associations with proliferation and metastasis‐related genes using publicly available datasets. However, several important limitations must be acknowledged. First, the analyses rely solely on transcriptomic data, and mRNA expression levels may not accurately reflect protein expression, post‐translational modifications, or enzymatic activity of C1GALT1, particularly given its critical role in O‐glycosylation. The presence of C1GALT1 mRNA does not guarantee the synthesis of functional core 1 O‐glycans in tumor tissues, especially without accompanying proteomics or glycomics validation.

Second, this study is entirely in silico and lacks experimental confirmation through in vitro or in vivo models, which limits the ability to infer causality from the observed statistical correlations. The positive or negative associations between C1GALT1 and immune cell infiltration, proliferation markers, and metastasis‐related genes should be interpreted as hypothesis‐generating observations rather than definitive biological relationships. Additionally, the study did not account for tumor subtype heterogeneity (e.g., molecular subtypes of breast or lung cancer), which may affect the interpretation of gene expression and immune associations. The analysis also assumes consistency in tumor purity and quality across datasets, which may introduce confounding variables. Another limitation is the lack of matched normal samples in some cancers, and small M1 subgroup sizes limited the power of metastatic analyses. Additionally, only DNA methylation was assessed, leaving out other epigenetic regulators (e.g., histone modifications, chromatin accessibility) that were not examined, which may also contribute to the regulation of C1GALT1 expression. Despite this, the study provides a foundation for future research into C1GALT1 as a potential biomarker and therapeutic target.

## 6 Conclusion

This bioinformatic analysis highlights the complex role of C1GALT1 in cancer, showing its differential expression, epigenetic regulation, and associations with prognosis, proliferation, metastasis, and immune modulation. C1GALT1 was upregulated in several GI and GU cancers, correlating with poor survival and elevated expression of proliferation markers like MKI67, PCNA, and MCM2–7, suggesting a role in tumor growth. Its expression inversely correlated with DNA methylation and was strongly co‐expressed with Cosmc, indicating coordinated regulation.

C1GALT1 also showed variable associations with immunosuppressive cells (Tregs, MDSCs), implying a context‐dependent role in shaping the immune microenvironment. However, these findings are based solely on transcriptomic data and lack functional validation. As mRNA levels may not reflect glycosylation activity, further in vitro and in vivo studies are needed to clarify its mechanistic role. While C1GALT1 shows potential as a biomarker or therapeutic target, its clinical relevance remains to be established.

## Author Contributions

**E.K.:** conceptualization, investigation, data curation, conceptualization, table and figure preparation, writing – original draft. **A.C.:** conceptualization, writing – review and editing, supervision.

## Ethics Statement

The authors have nothing to report.

## Consent

The authors have nothing to report.

## Conflicts of Interest

The authors declare no conflicts of interest.

## Supporting information


**Figure S1.** C1GalT1 gene expression differences between normal and tumor samples across cancer types by using the TIMER2.0 portal.
**Figure S2.** Overall survival (OS) comparison between high and low C1GalT1 gene expression groups displayed on KM‐plots across cancer types was not statistically significant.

## Data Availability

The data that support the findings of this study are available from the corresponding author upon reasonable request.
