# Supplementary material for: Bioinformatic Analysis of C1GALT1 in Cancer: Insights Into Prognosis, Metastasis and Therapeutic Potential
Source: Cancer Rep (Hoboken). 2025 Jun 23;8(6):e70259. doi: 10.1002/cnr2.70259 (PMC12183607; doi:10.1002/cnr2.70259)

**The Role of C1GALT1 in Cancer: Insights into Glycosylation, Prognosis, and Therapeutic Potential**

Ecem Kalemoglu ^1,2*^, Ayse Caner^2,3,4*^

^1^ Department of Internal Medicine, Rutgers-Jersey City Medical Center, 355 Grand St, Jersey City, NJ, USA, 07302.

^2^ Department of Basic Oncology, Institute of Health Sciences, Ege University, Izmir, Turkey.

^3^ Translational Pulmonary Research Center (EGESAM), Ege University, Izmir, Turkey.

^4^ Department of Parasitology, Faculty of Medicine, Ege University, Bornova, 35100, Izmir, Turkey.

^*^Corresponding authors:

Ecem Kalemoglu, Department of Internal Medicine, Rutgers University-Jersey City Medical Center, 355 Grand St, Jersey City, NJ, USA, 07302

E-mail: [ecem.kalemoglu@rutgers.edu](mailto:ecem.kalemoglu@rutgers.edu); ecemkalemoglu@gmail.com.

Phone: +1 404 716 03 85

ORCID ID: 0000-0002-6312-0048

Ayse Caner, Department of Basic Oncology, Faculty of Medicine, Ege University, Bornova, 35100, Izmir, Turkey.

E-mail: ayse.caner@ege.edu.tr; ayecaner@gmail.com

Phone: +90 505 485 59 94

ORCID ID: 0000-0003-3058-9971

**Supplemental Material**

1. **Supplement Figure 1.** C1GalT1 gene expression differences between normal and tumor samples across cancer types by using the TIMER2.0 portal.


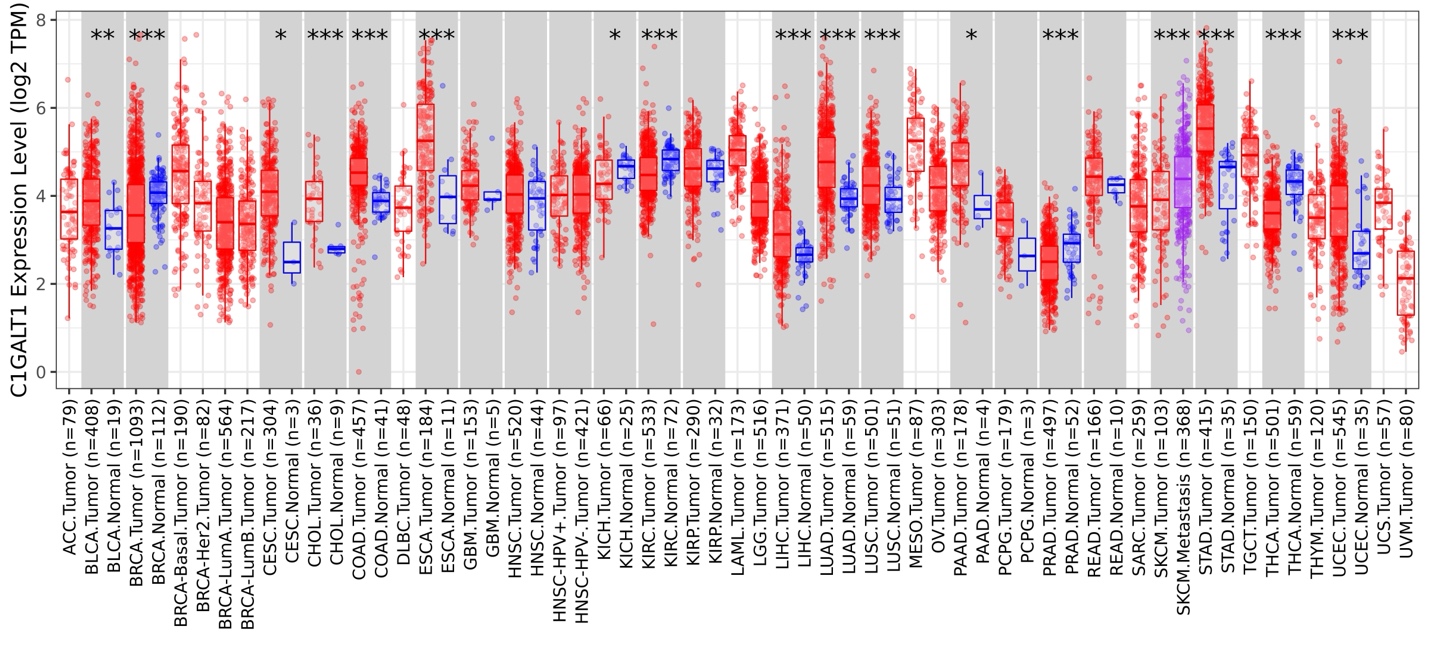


ACC: Adrenocortical Carcinoma; BLCA: Bladder Urothelial Carcinoma; BRCA: Breast Invasive Carcinoma; CESC: Cervical and Endocervical Cancer; CHOL: Cholangiocarcinoma; COAD: Colon Adenocarcinoma; DLBC: Diffuse Large B-cell Lymphoma; ESCA: Esophageal Carcinoma; GBM: Glioblastoma Multiforme; HNSC: Head and Neck Cancer; KICH: Kidney Chromophobe; KIRC: Kidney Renal Clear Cell Carcinoma; KIRP: Kidney Renal Papillary Cell Carcinoma; LAML: Acute Myeloid Leukemia; LGG: Lower Grade Glioma; LIHC: Liver Hepatocellular Carcinoma; LUAD: Lung Adenocarcinoma; LUSC: Lung Squamous Cell Carcinoma; MESO: Mesothelioma; OV: Ovarian Serous Cystadenocarcinoma; PAAD: Pancreatic Adenocarcinoma; PCPG: Pheochromocytoma and Paraganglioma; PRAD: Prostate Adenocarcinoma; READ: Rectum Adenocarcinoma; SARC: Sarcoma; SKCM: Skin Cutaneous Melanoma; STAD: Stomach Adenocarcinoma; TGCT: Testicular Germ Cell Tumors; THCA: Thyroid Carcinoma; THYM: Thymoma; UCEC: Uterine Corpus Endometrial Carcinoma; UCS: Uterine Carcinosarcoma; UVM: Uveal Melanoma; (*: p-value < 0.05; **: p-value <0.01; ***: p-value <0.001).

1. **Supplement Figure 2.** Overall survival (OS) comparison between high and low C1GalT1 gene expression groups displayed on KM-plots across cancer types was not statistically significant.


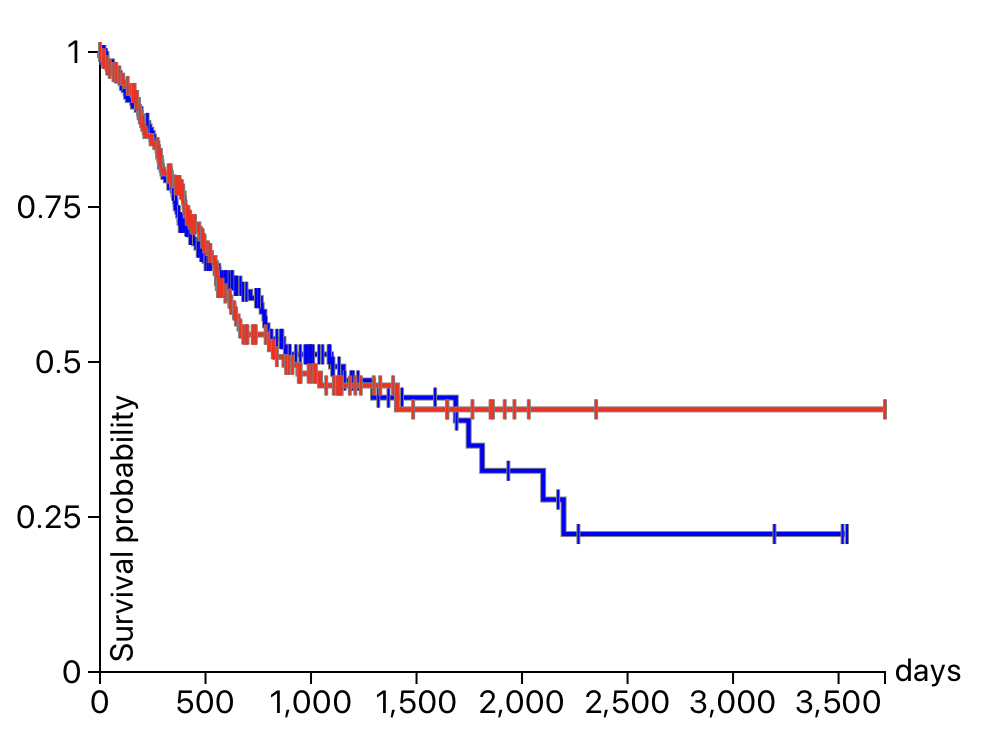

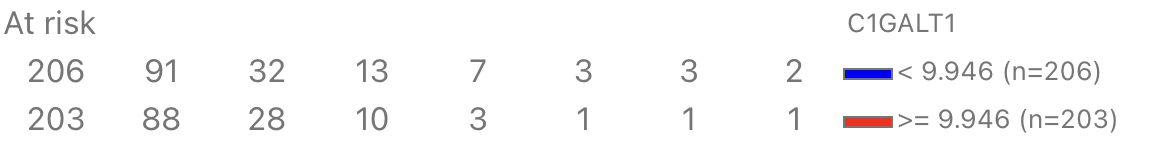


*p* = 0.9118

Stomach Cancer


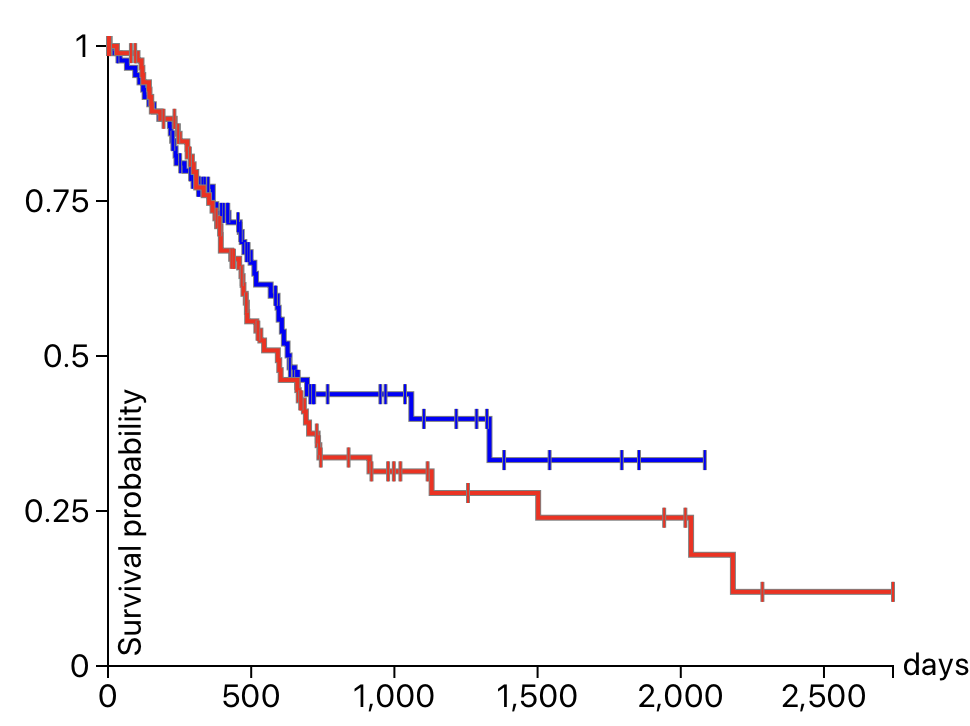

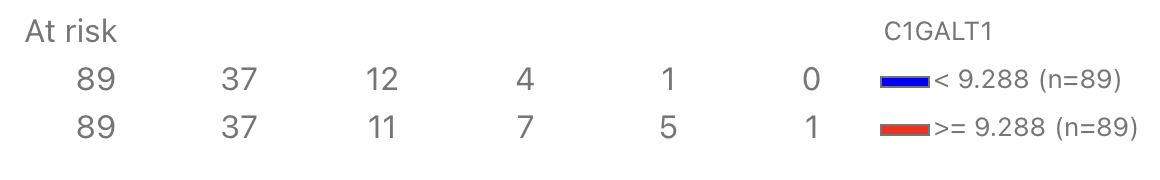


Pancreatic Cancer

*p* = 0.3430

Colon and Rectal Cancer


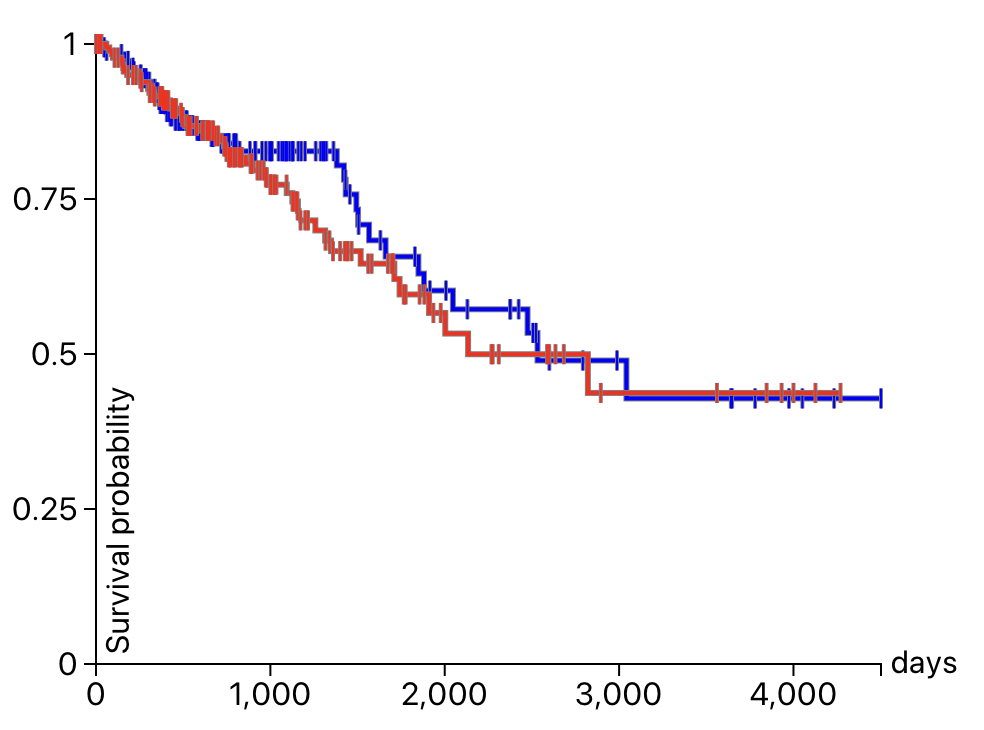

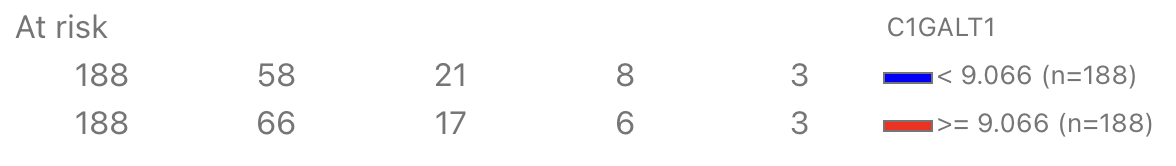


*p* = 0.5490


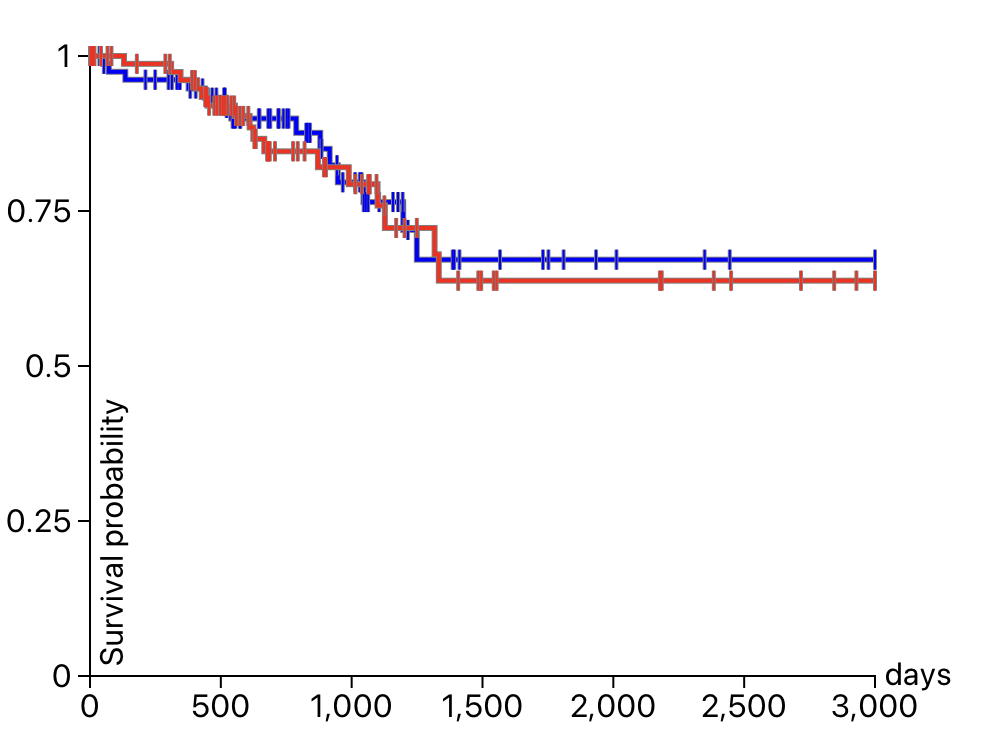

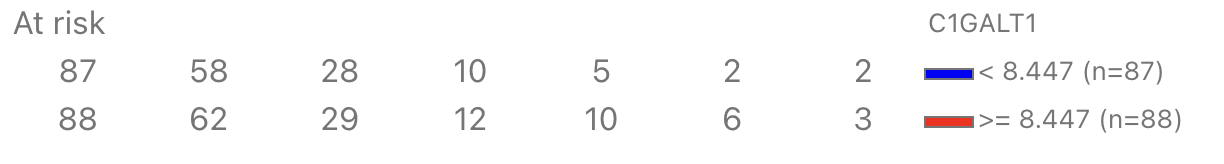


*p* = 0.8237

Endometrioid Cancer


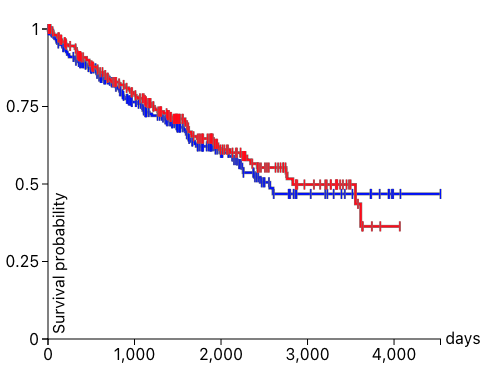

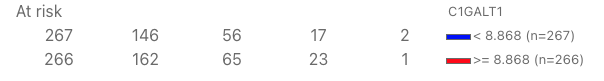


*p* = 0.5816

Kidney Clear Cell Carcinoma


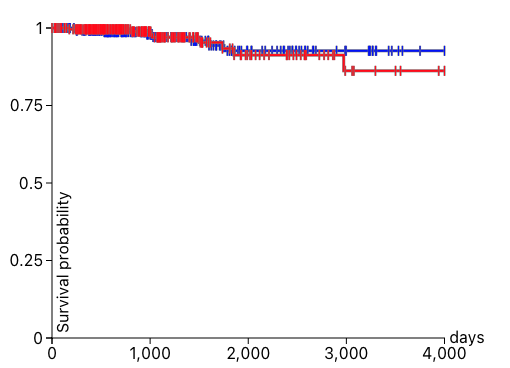

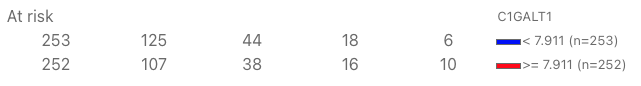


Thyroid Cancer


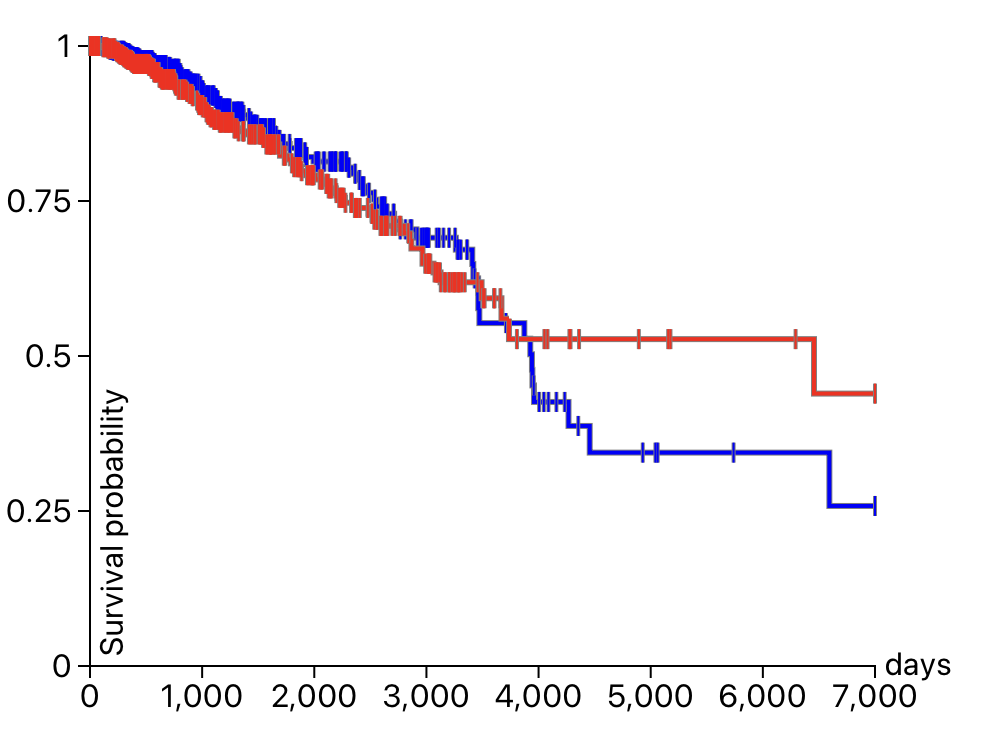

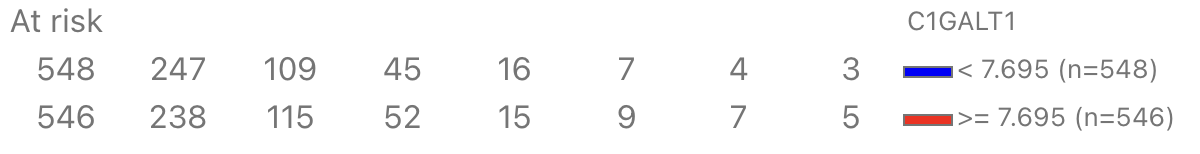


*p* = 0.6142

Breast Cancer

*p* = 0.8014


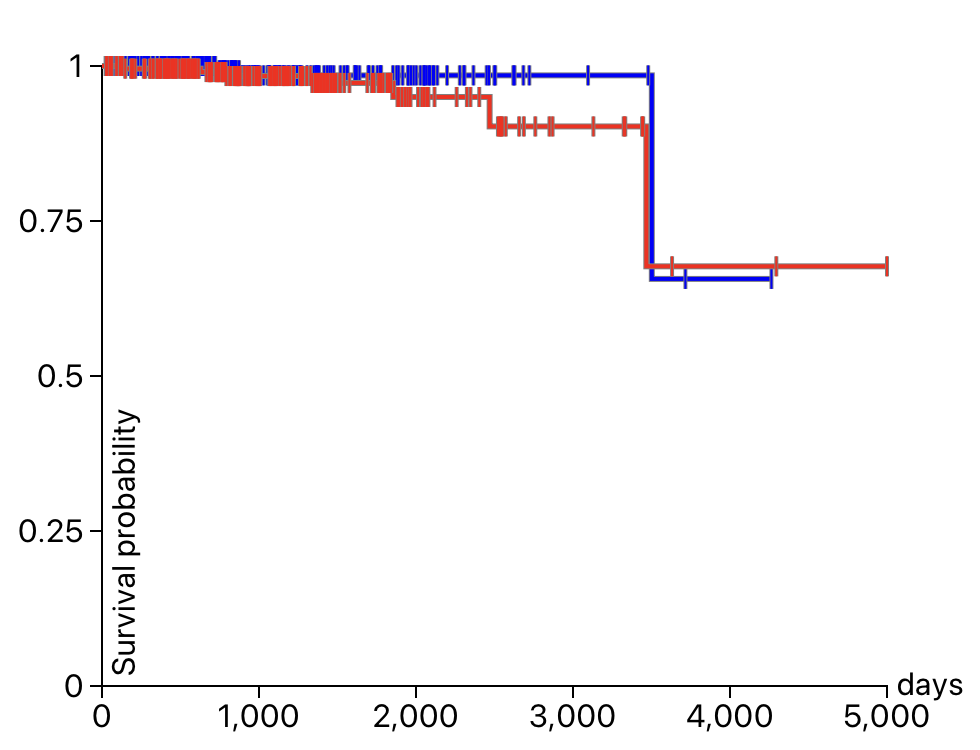

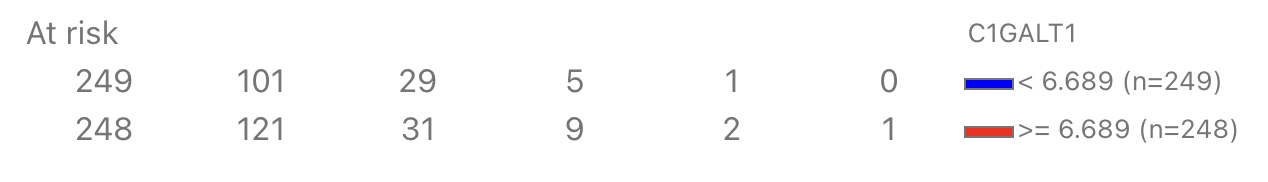


*p* = 0.3090

Prostate Cancer


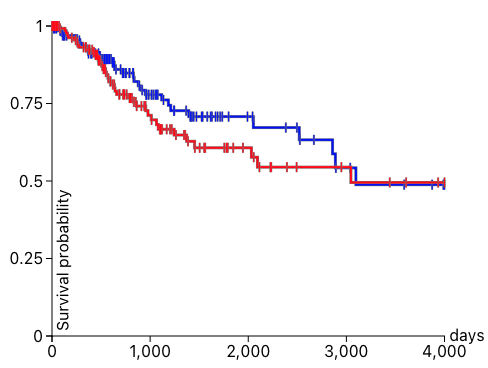

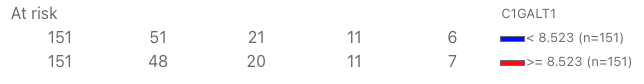


*p* = 0.2911

Cervical Cancer


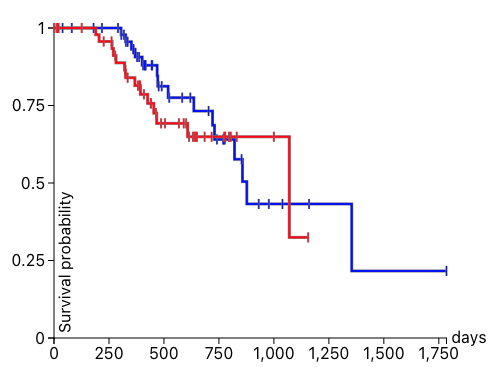

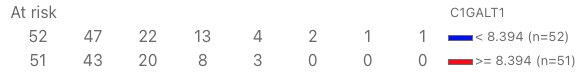


*p* = 0.4636

Melanoma


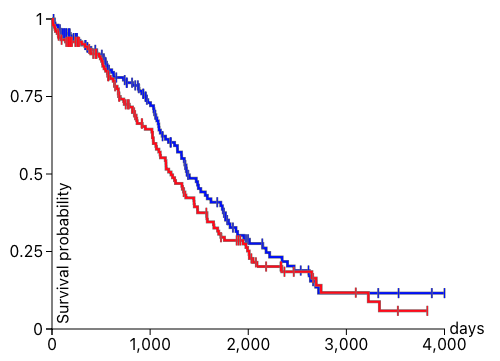

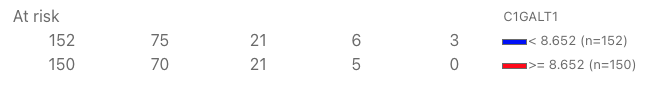


*p* = 0.2195

Ovarian Cancer

*p* = 0.06499

Glioblastoma


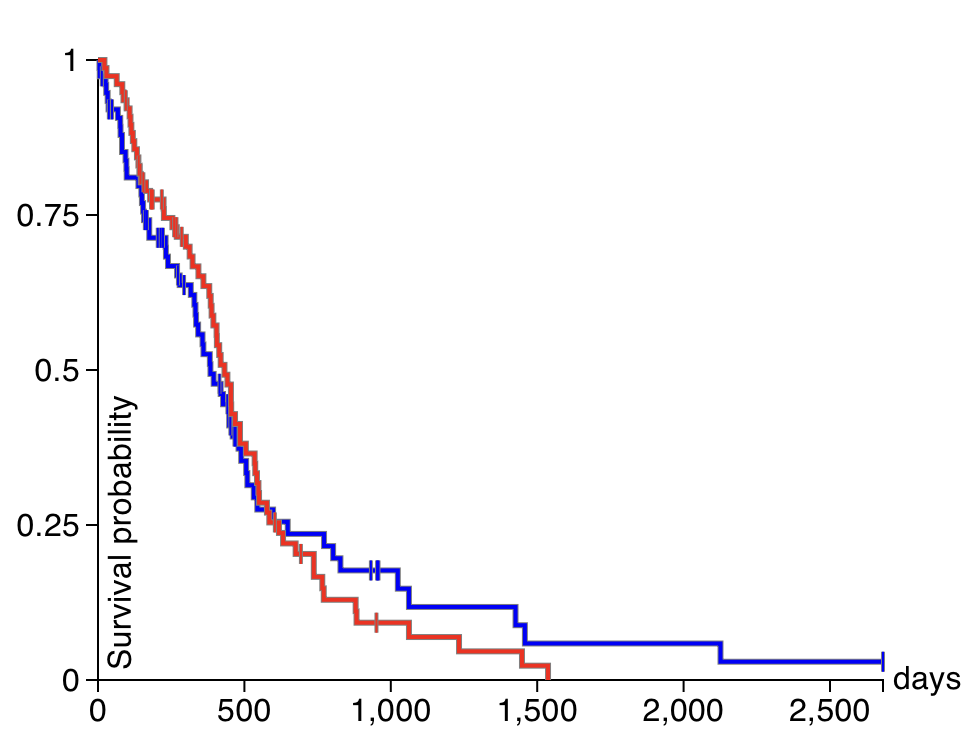

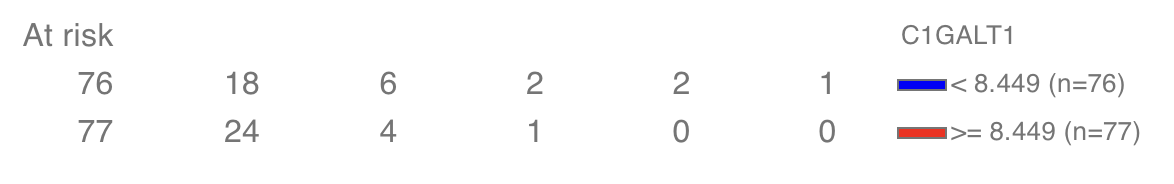

Supplement: Supplementary file 1 — Figure S1. C1GalT1 gene expression differences between normal and tumor samples across cancer types by using the TIMER2.0 portal. Figure S2. Overall survival (OS) comparison between high and low C1GalT1 gene expression groups displayed on KM‐plots across cancer types was not statistically significant. [file CNR2-8-e70259-s001.docx]
